# Transcriptome profiling of esophageal squamous cell carcinoma reveals a long noncoding RNA acting as a tumor suppressor

**DOI:** 10.18632/oncotarget.4185

**Published:** 2015-05-19

**Authors:** Guifeng Wei, Huaxia Luo, Yu Sun, Jiagen Li, Liqing Tian, Wei Liu, Lihui Liu, Jianjun Luo, Jie He, Runsheng Chen

**Affiliations:** ^1^ Bioinformatics Laboratory and CAS Key Laboratory of RNA Biology, Institute of Biophysics, Chinese Academy of Sciences, Beijing, China; ^2^ University of Chinese Academy of Sciences, Beijing, China; ^3^ Department of Thoracic Surgery, Cancer Institute and Hospital, Chinese Academy of Medical Sciences and Peking Union Medical College, Beijing, China; ^4^ Research Network of Computational Biology, RNCB, Beijing, China

**Keywords:** ESCC, long noncoding RNAs, tumor suppressor, metastasis, Epist

## Abstract

Esophageal Squamous Cell Carcinoma (ESCC) is among the most common malignant cancers worldwide. In the past, extensive efforts have been made to characterize the involvement of protein-coding genes in ESCC tumorigenesis but few for long noncoding RNAs (lncRNAs). To investigate the transcriptome profile and functional relevance of lncRNAs, we performed an integrative analysis of a customized combined lncRNA-mRNA microarray and RNA-seq data on ESCCs and matched normal tissues. We identified numerous lncRNAs that were differentially expressed between the normal and tumor tissues, termed “ESCC-associated lncRNAs (ESCALs)”, of which, the majority displayed restricted expression pattern. Also, a subset of ESCALs appeared to be associated with ESCC patient survival. Gene set enrichment analysis (GSEA) further suggested that over half of the ESCALs were positively- or negatively-associated with metastasis. Among these, we identified a novel nuclear-retained lncRNA, named *Epist*, which is generally highly expressed in esophagus, and which is down-regulated during ESCC progression. *Epist* over-expression and knockdown studies further suggest that *Epist* inhibits the metastasis, acting as a tumor suppressor in ESCC. Collectively, our analysis of the ESCC transcriptome identified the potential tumor suppressing lncRNA *Epist*, and provided a foundation for future efforts to identify functional lncRNAs for cancerous therapeutic targeting.

## INTRODUCTION

Human esophageal cancer is one of the most deadly cancers, ranking as the sixth leading cause of cancer-related deaths worldwide [1]. In China, esophageal squamous cell carcinoma (ESCC) is the major subtype of esophageal cancer, accounting for over 90% of the cases [2]. Despite improvements in diagnostic techniques and therapeutic modalities, ESCC still remains a devastating malignancy, mainly due to the late diagnoses and the aggressive features of the disease. As with other cancers, the pathogenesis of ESCC appears to result from the dysregulation of protein-coding and noncoding genes involved in a number of vital functions such as cell cycle control, cell differentiation, cell migration, invasion and other cancer-related pathways. Recent studies of ESCC cell lines and clinical samples have suggested that epigenetic silencing, *e.g.* DNA methylation, of key tumor suppressor genes is critical to the initial and progressive steps of ESCC [3, 4]. However, the detailed mechanisms leading to ESCC have not yet been fully elucidated. Identification of aberrantly expressed coding genes as well as noncoding RNAs may be crucial steps for both the diagnosis of ESCC and subsequent personalized treatment. Recently, a number of protein-coding genes, such as *PTK6* and *Rab25*, have been identified as having tumor suppressor activity through modulating different signaling pathways [5, 6]. High levels of DNA methylation at the promoter regions of these genes in ESCC cell lines are common observations [3]. Several microRNAs have also been linked to various aspects of ESCC, such as promotion of cell migration and invasion (*microRNA-25*) or reduction of the cell cycle (*miR-29c*), thus being potential biomarkers of this disease [7, 8].

Long noncoding RNAs (lncRNAs) are identified as non-protein-coding transcripts, which are longer than 200 nt and are often spliced, 3′-polyadenylated and have a 7-methylguanosine cap at 5′-terminal. LncRNAs are believed to be implicated in diverse biological processes and disease-related pathways in both vertebrates and invertebrates, controlling the aspects of processes such as cellular proliferation, development, lineage commitment, immune response, pluripotency and differentiation [9-13]. The identification of lncRNAs has expanded our notions of the transcriptome complexity and gene regulatory network. Although many efforts have been made to annotate the lncRNAs encoded in the human genome, the functions of most lncRNAs are still uncertain. And only a few dozen of the lncRNAs have been well characterized to date. For example, *HOTAIR* [14] and *ANRIL* [15] facilitate gene repression through recruiting the Polycomb Repressive Complex 2 (PRC2) to change the epigenetic state of their target genes, the *megamind* and *cyrano* lincRNAs play essential roles in the organogenesis of zebrafish embryos [11], and the lincRNA, *Fendrr* is involved in the regulatory networks controlling the fate of lateral mesoderm derivatives [16].

In recent years, accumulating evidence have suggested that an increasing number of reports have shown that lncRNAs play key roles in cancer and diseases [17-20]. Studies have associated the aberrant expression of lncRNAs with the tumor progression or metastasis, for example, *PCAT-1* and *SChLAP1* in prostate cancer [17, 18], *HOTAIR* in breast [20] and colorectal cancer [21]. However, there are still no comprehensive reports focusing on the functional roles of lncRNAs in ESCC. Very recently, an expression profile study on a large cohort of ESCC patients revealed an association between lncRNA expression level alterations and the clinical outcome for ESCC patients [22]. Besides, other studies have indicated that lncRNA expression shows higher tissue-specificity than that of protein-coding gene [23], suggesting that lncRNAs may have advantages as biomarkers for ESCC. Thus identifying ESCC related lncRNAs and their functions might provide valuable insights into the pathogenesis of ESCC as well as potential biomarker candidates.

In the present study, we implemented the integrative analysis of the long noncoding transcriptome of ESCCs and patient-matched normal specimens using a customized microarray and public RNA-seq data, thereby identifying a number of transcripts differentially expressed in normal and tumor tissues. Furthermore, we systematically assessed the tissue specificity of the lncRNA expression and its association with patient survival. Also, the GSEA method was applied to analyze the involvement of the ESCC associated lncRNAs in three biological processes (cell cycle, metastasis and apoptosis). Finally, we experimentally validated the functional role (metastasis) for one of the ESCALs, termed *Epist*, which acts as a tumor suppressor in ESCC tumorigenicity.

## RESULTS

### The long noncoding transcriptome profile of ESCC

Esophageal Squamous Cell Carcinoma (ESCC) is one of the most deadly forms of cancer across the world. In order to investigate the relationship between lncRNAs expression and ESCC, we reanalyzed the microarray data from pairs of ESCC and matched surrounding normal tissues of 119 patients [22] in combination with RNA-seq datasets [5, 6] (see Materials and Methods for details). We re-annotated the probes in the original microarray data (Figure S1A), and the result being that 11629 lncRNAs were interrogated by one or more unique probes (see Li *et al*. [22]; Materials and Methods; Table S1).

**Figure 1:**
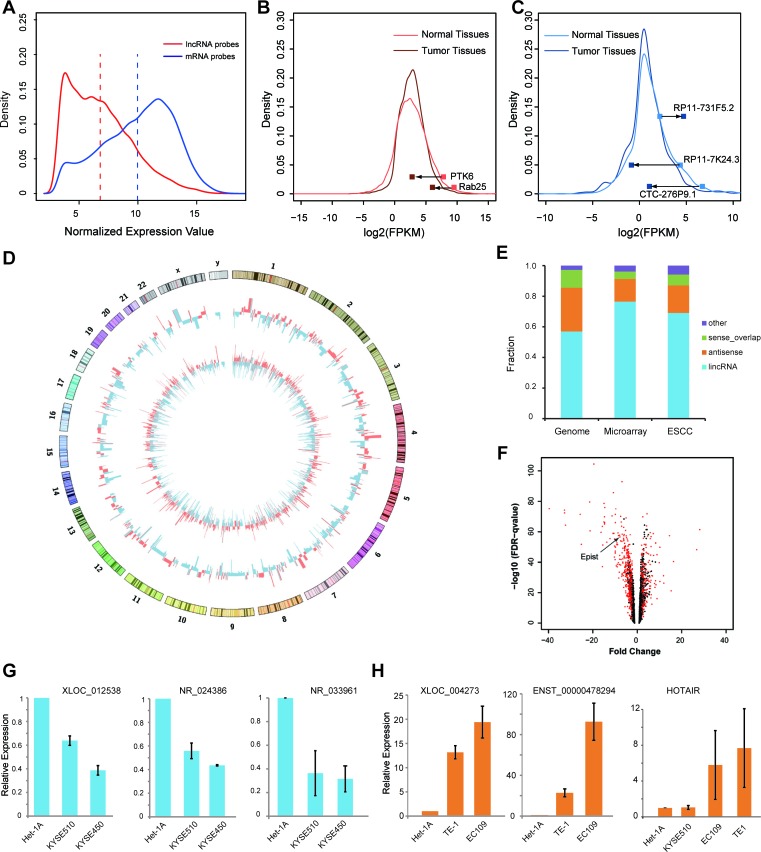
Transcriptome analysis of long noncoding RNAs in esophageal squamous cell carcinoma (ESCC) **A.** Distribution of the expression levels of long noncoding RNAs (red) and protein-coding genes (blue) represented in the combined microarray. Dashed lines indicates the average value (6.799 for lncRNA and 9.963 for protein-coding genes). **B.**, **C.** The density plot of the abundance of protein-coding genes **B.** and lncRNAs **C.** (log2-RPKM determined by Cufflinks) in the RNA-seq data. Several recently identified tumor suppressor genes (PTK6, Rab25) and lncRNAs that were differentially expressed between ESCC and adjacent normal tissues are indicated. **D.** The Circos plot showing genome-wide differential expression of long noncoding RNAs (930 probes) and mRNAs (3220 probes) between ESCCs and matched normal tissues. The outer and inner tracks indicate the lncRNAs and mRNAs, respectively (red, up-regulated; blue, down-regulated). The heights of the bars indicate fold differences. **E.** The barplot shows the biotype fraction of genome-scale, microarray assayed, differentially expressed lncRNAs (long intergenic noncoding RNA, Antisense, Sense_overlap and other biotypes). **F.** The volcano plot showing all the lncRNA probes assayed in the microarray and the differentially expressed lncRNAs (ESCALs) are indicated by the red dots. (The arrow suggests *Epist*). **G.**, **H.** Quantitative RT-PCR analysis of several selected ESCALs in cell lines. **G.** Down-regulated ESCALs (*XLOC_012538, NR_024386* and *NR_033961*); **H.** Up-regulated ESCALs (*XLOC_004273, ENST00000478294* and *HOTAIR*). Het-1A is an immortalized normal esophageal epithelial cell line; TE-1, EC109, KYSE510 and KYSE450 are ESCC cell lines. Error bars, S.E.M.

Compared with protein-coding genes, the expression levels of lncRNAs were markedly lower in ESCC tissues (*p*-value < 2.2e-16; Figure 1A). The expression of most (~90%) of the annotated lncRNAs was also extremely low or not detected in the RNA-seq data (GSE29968). Specifically, 12.58% (1745) of the 13870 GENCODE (v19) annotated long noncoding RNAs were detected, and similarly, only 6.07% (498) of 8195 lincRNAs from Cabili *et al.* [23] were detected. By contrast, approximately 70% of the RefSeq genes were robustly detected. These differences are in accordance with previous reports that the expression levels of lncRNAs are generally lower than those of protein-coding genes [23] (Figure 1B-1C).

Recently, the *Rab25* and *PTK6* genes were identified as tumor suppressors in ESCC, and these two genes were markedly down-regulated in ESCC tumorigenesis both in previous [5, 6] and in this study (Figure 1B). Next, we called the aberrantly expressed protein-coding genes across this cohort of ESCC patients (Materials and Methods; Table S2), and Gene Ontology enrichment analysis of protein-coding genes with aberrant expression in ESCC and normal tissues indicated that a number of biological processes were enriched, such as cell cycle, epithelial cell differentiation (Figure S2). Similarly, there were also numerous lncRNAs that were markedly up- or down-regulated during the ESCC progression (Figure 1C). To further investigate this, we integrated the above microarray and RNA-seq data to interrogate the differentially expressed lncRNAs between ESCC and adjacent normal esophageal tissues.

### LncRNAs are differentially expressed in ESCC relative to adjacent normal tissues

To determine how many lncRNAs were differentially expressed and have the potential roles during the cancer-related processes, the expression detected at each lncRNA probe was screened by a stringent criterion (as described in Methods). Therefore, of the 13589 probes interrogating lncRNAs, 930 probes (representing 827 lncRNAs) were considered significantly differentially expressed in the microarray analysis, including 341 and 589 probes showing up-regulated and down-regulated expression, respectively (Figure 1D-1F). The differentially expressed lncRNAs corresponding to these probes were termed as “ESCC associated lncRNAs (ESCALs)”. And most of ESCALs belong to groups on lncRNAs previously annotated “lincRNAs” and “antisense RNAs” (Figure 1E). To further confirm the observed differences in expression, we randomly selected a subset of ESCALs and used qRT-PCR to determine their expression levels in an immortalized normal esophageal epithelial cell line (Het-1A) and in several ESCC cell lines (Figure 1G-1H).

Besides, we also explored other two public RNA-seq data to profile the long noncoding transcriptome of ESCC and adjacent normal tissues (Figure S3A). Given the characteristics of the RNA-seq data, we adopted distinct methods for calling the differentially expressed genes (Document S1 and Figure S3). And the differentially expressed genes called by these two RNA-seq data were shown in Table S3. In addition, we also searched for novel transcripts and fusion transcripts, the methods and results were stated in Document S3.

### Aberrantly expressed lncRNAs (ESCALs) across human cancers

Next, we asked whether the identified ESCALs have tissue specific expression patterns, or potential to be as biomarkers. To this end we conducted the analysis of comparing the ESCALs with the lncRNAs expression profile in diverse cancers.

We interrogated the transcriptional profile among 64 archived human cancers comprising 17 diagnostic subtypes of squamous cell carcinomas (head and neck, lung, except esophagus), adenocarcinoma as well as sarcomas [19]. Only 190 (~23%) out of the 827 ESCALs were expressed as assessed by 3SEQ (3′-End Sequencing for Expression Quantification) in these tumor samples, and even a smaller fraction (112, ~13.5%) of the ESCALs were also observed as differentially expressed across the panel of assayed tumor samples (Table S4A-S4B). One particular case is the lncRNA, *MALAT-1* [24], which is generally among the most highly expressed lncRNAs. *MALAT1* shows no marked differences in expression between tumors and normal surrounding tissues, suggesting that this lncRNA is not associated with the tumorigenicity of ESCC. The same applies to some other well-characterized lncRNAs, such as *ANRIL* [15].

We also intersected the ESCALs with the LncRNADisease database [25], which has collected and curated hundreds of lncRNAs associated with diverse human diseases. However, only 19 long noncoding RNAs from the LncRNADisease were found among the ESCALs, these including several lncRNAs such as *HOTAIR*, *PVT1*, *H19* and *GAS5* that have been reported to be dysregulated in multiple human cancers. Among these, the expression of *H19* and *HOTAIR* was validated in the Het-1A and ESCC cell lines by qRT-PCR. *HOTAIR*, binds physically to the chromatin modifying enzymes PRC2 and LSD1 to reprogram the chromatin state and further promote cancer metastasis in breast cancers [20] and colorectal cancers [21], also exhibits oncogenic activity in ESCC [26]. Interestingly, two long noncoding RNAs associated with barrett's esophagus or squamous carcinoma, *AFAP1-AS1* and *UCA1*, respectively, were also included. There may be some suggestions that these lncRNAs play the similar functional roles in ESCC tumorigenesis as previously reported (Table S4C).

All the results indicated that the majority of ESCALs showed restricted expression patterns to esophagus epithelial cell or esophageal squamous cell carcinomas (ESCC) and there may be other uncharacterized lncRNAs important during the ESCC progression.

### LncRNAs expression and ESCC patient survival

**Figure 2:**
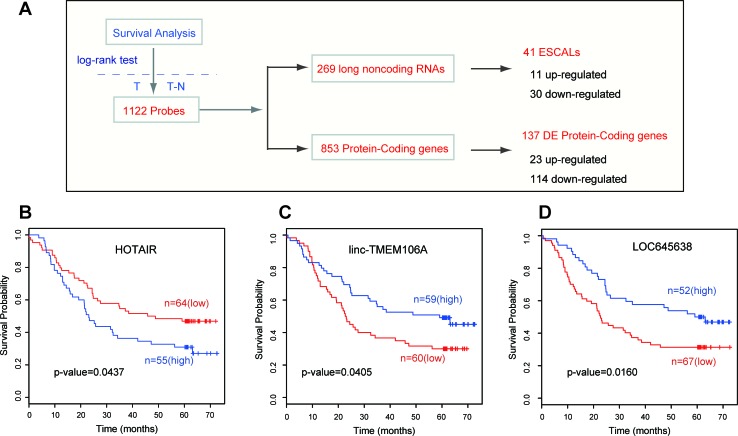
Long noncoding RNAs associated with survival time of ESCC patients **A.** The workflow of screening the mRNA and lncRNA probes whose expression level associates the survival time of ESCC patients, as described in Methods. (**B**, **C**, **D**) These panels show the Kaplan-Meier survival curves for three lincRNA examples of them. *HOTAIR, linc-TMEM106A* and *LOC645638*.

Our previous study identified a three-lncRNA signature, which might serve as a new biomarker for the prognosis of ESCC patients [22]. To further explore the relationship between the expression levels of certain lncRNAs and the survival time of ESCC patients, we performed a log-rank survival analysis for every coding and noncoding probe assayed in microarray (Materials and Methods; Table S5). A total of 1122 probes showed significant association with ESCC patient survival, including the mRNA, Rab25 [6] and lincRNA, *HOTAIR* [26] previously reported from other groups based on different cohorts of ESCC patients (Figure 2A). Among the 827 ESCALs, 41 were significantly (log rank test, *p*-value<0.05) associated with the survival of ESCC patients. Of which, 11 were up-regulated and 30 down-regulated in tumor tissues. Figure 2B-2D showed the Kaplan-Meier survival curves across the 119 ESCC patients for the up-regulated *HOTAIR* and two down-regulated ESCALs (*linc-TMEM106A* and *LOC645638*). Collectively, this suggests that the expression levels of lncRNAs can also be used for survival prediction of ESCC patients.

### Expression of lncRNAs is associated with metastasis, cell cycle and apoptosis

**Figure 3:**
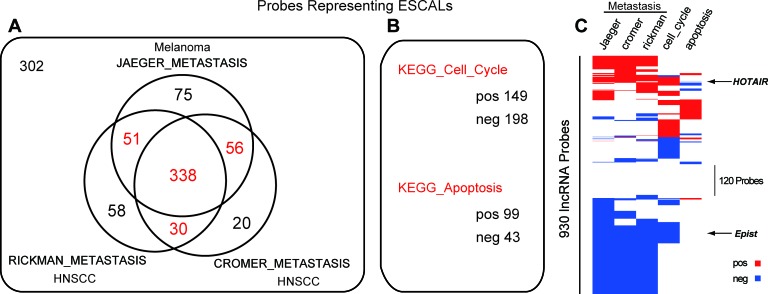
Long noncoding RNAs associated with metastasis, cell cycle and apoptosis **A.** Venn diagram of ESCALs probes associated with three distinct metastatic gene sets (Jaeger's melanoma study [54] and Rickman's and Cromer's head and neck squamous cell carcinoma study [55, 56]). **B.** Number of probes representing ESCALs, which were positively- and negatively-correlated with KEGG_CELL_CYCLE and KEGG_APOPTOSIS gene collections. **C.** The heatmap showing the hierarchical clustering of 930 probes of ESCALs associated with metastasis, cell cycle and apoptosis gene sets. Red, white, blue indicate positive, no and negative association, respectively.

It is well described that, deregulation of cell cycle, apoptosis and metastasis are critical in tumorigenesis. However, little is known about how and how many lncRNAs are involved in these biological processes. To try to address this question, we applied gene set enrichment analysis (GSEA) to evaluate the potential association of the ESCALs in cancerous biological processes (cell cycle, apoptosis, metastasis), using the relative difference (fold change) in lncRNA expression (ΔE = T - N) and correlation with that of protein-coding genes (Materials and Methods; Table S6). To assess the validity of the predicted functional associations, we examined the enriched gene sets for the lncRNA, *HOTAIR*. The GSEA result showed that *HOTAIR* positively correlates with the metastasis gene sets (Figure 3A, 3C), which is consistent with the known properties of promoting metastasis [20]. Consequently, we found that more than half of the probes representing ESCALs (475 out of 930 probes) were functionally associated with metastasis, and one third associated with cell cycle processes, while only ~15% with apoptosis (Figure 3B-3C). Thus, in all ~87% of the ESCALs may be involved in one or more cancerous pathways, while ~13% of them could not be associated with any of these three biological processes, indicating they may be involved in other processes of tumorigenesis, such as tumor growth pathway. Taken together, these results indicate that the ESCALs may play critical roles during the ESCC tumorigenesis.

### Molecular characterization and expression pattern of *Epist*

**Figure 4:**
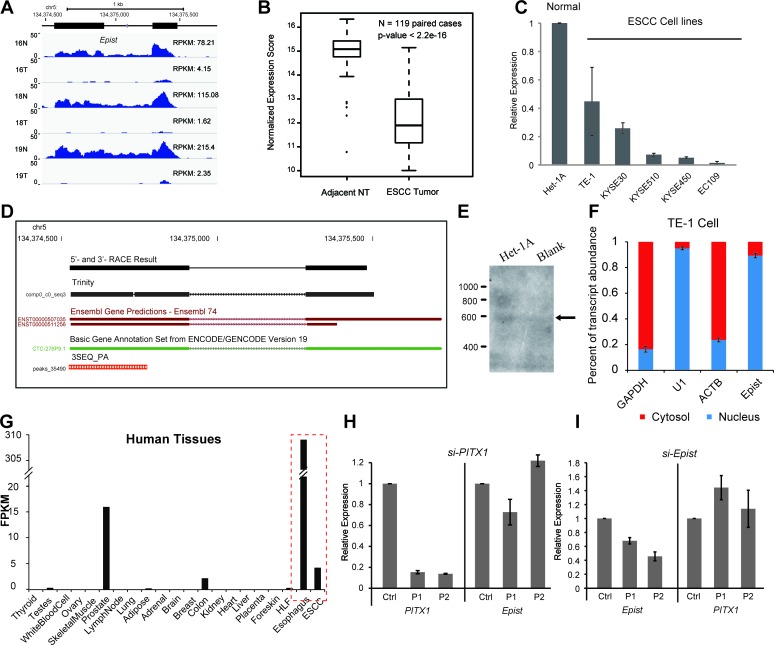
Genomic features and expression pattern of *Epist* **A.**,**B.**,**C.** The expression of *Epist* in tumor tissues or cancer cell lines was decreased compared to adjacent normal tissues or an esophageal epithelial cell line, respectively. **A.** RNA-seq data **B.** Microarray data assayed 119 ESCC paired tissues **C.** Immortalized normal esophageal epithelial cell line (Het-1A) and several ESCC cell lines (TE-1, KYSE510, KYSE450, KYSE30, EC109). **D.** Genomic features of *Epist*. RACE and Trinity results are shown. The 3SEQ_PA track is from Brunner *et al*. [19]. **E.** Northern Blot of Epist in total RNAs from Het-1A cells or blank control. **F.** Cellular fractionation was performed in TE-1 cells followed by RNA isolation and qRT-PCR, demonstrates that *Epist* is a nuclear-retained lincRNA. GAPDH and ACTB are cytoplasmic control and U1 servers as the nuclear control. Error bars indicate S.E.M. **G.** RNA-seq abundance estimates across a panel of adult human tissues. The data were retrieved from human body map project. FPKM, fragments per kilo bases of transcript per million mapped reads. The dash lines denote the esophageal tissues. (H,I) siRNA-mediated knock-down of PITX1 and *Epist*. **H.** Knockdown of PITX1 shows no significant impact on the expression of *Epist*. **I.** Knockdown of *Epist* leads to no remarkable alteration of PITX1 expression.

To explore the functional significance of lncRNAs in ESCC, we focused our attention on a number of ESCALs which were relatively highly expressed and for which GSEA suggested associations with a specific biological function. For these ESCALs, we calculated a tissue-specificity score and identified one transcript which was significantly (>=2-fold) down-regulated in the tumors in almost all patients (107/122, ~88%) (Figure 4A-4C and Figure 1F), and which was specifically highly expressed in normal esophagus epithelial cells indicated by the RNA-seq data (Figure 4G) and epigenetic landscape [27] (Figure S4). Then we termed this transcript, esophagus epithelial intergenic specific transcript, or *Epist*. Performance of 5′- and 3′-RACE and Northern Blot determined *Epist* as an ~600 nt long transcript, originating from a locus of approximately 1 kb in chromosome 5. Additionally, 3SEQ-Seq analysis further showed that a poly-A signal occurred at ~4.5kb upstream of the neighbouring protein-coding gene, PITX1 [19]. Although the transcriptional orientation of *Epist* is the same as its nearby genes, the RACE results and the presences of a poly-A signal indicated that *Epist* is an independent transcription unit rather than the extension of 5′-UTR of PITX1. Also, we used the Cufflinks and Trinity software to assemble the gene structure of *Epist*. Both the computational and experimental results suggested that *Epist* is an approximately 600-nt transcript consisting of two exons (Figure 4D-4E).

To characterize the functional relevance of *Epist*, we firstly addressed whether the transcript is indeed noncoding. The computational analysis of coding potential suggested that the *Epist* sequence has a very low coding potential (Document S2). Secondly, isolation of the nuclear and cytosolic fractions of TE-1 cell showed that the *Epist* transcript was mainly located in the nucleus (similar to that of U1, Figure 4F).

Further, we examined the expressional relationship between *Epist* and PITX1. The expression of *Epist* was highly correlated (R=0.95) with that of PITX1 across the cohort of ESCC patients (Figure 6A), indicating that there is an underlying connection between *Epist* and PITX1. However, we observed that knock-down of *Epist* could not significantly alter the expression of PITX1, and vice versa (Figure 4H-4I). These results showed that *Epist* does not directly regulate the transcription of PITX1 and vice versa, suggesting that *Epist* may exert its function in *trans*.

### *Epist* inhibits migration and invasion

**Figure 5:**
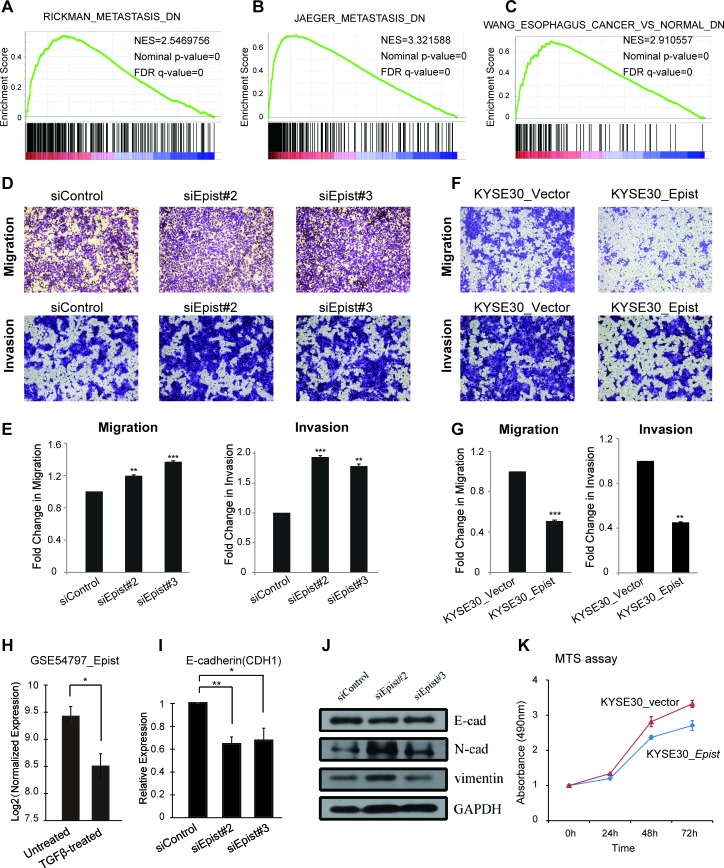
*Epist* inhibits migration and invasion processes **A.**, **B.** *Epist*-correlated genes are enriched in the gene signatures of metastasis from *Jaeger et al.* and *Rickman et al.* **C.** GSEA result shows *Epist*-correlated genes enriched in the gene set of previously identified differentially expressed genes in esophagus cancer. **D.** Representative images of transwell migration (top) and invasion (bottom) assays in siRNA-mediated knock-down of *Epist*. **E.** The barplot showed the summary of the number of migrated (left) and invaded (right) cells per field. **F.** Representative images of transwell migration (top) and invasion (right) assays in KYSE30 cells stably overexpressing *Epist* or control vector sequence. **G.** The barplot showed the summary of the number of migrated (left) and invaded (rigth) cells. **H.** *Epist* was down-regulated in the TGF-β-treated cells (GSE54797). **I.**, **J.** The mRNAs **I.** and protein levels **J.** of several EMT markers (E-cadherin, N-cadherin, Vimentin) after siRNA-mediated loss-of-function of *Epist* in KYSE30 cell line. **K.** Comparison of the cell proliferation measured by MTS assay for KYSE30 overexpressing *Epist* or control vector cell lines. All these data show the mean ± S.D. from three independent assays. Statistical significance was determined by Student's *t* test. *p < 0.05, **p < 0.01; ***p < 0.001.

According to the above GSEA result, genes that are expressionally correlated with *Epist* are also enriched for functions related to metastasis discovered by three independent studies (Figure 5A-5B; Materials and Methods; Figure S6A). Also, the previously down-regulated gene set of esophageal cancer (MSigDB) was significantly enriched in *Epist*-positively-associated genes [28] (Figure 5C). To study this further, we performed the transwell migration assays using the KYSE30 cell line, which has a higher expression of *Epist* than other cell lines (Figure 4C). The results showed that knock-down of *Epist* lead to an increased number of cells migrating through the membrane (Figure 5D). Next, we established a KYSE30 cell line which stably over-expressed the *Epist* lncRNA (Materials and Methods; Figure S5). Overexpression of *Epist* in the KYSE30 cells markedly reduced the effect of the migration as assessed by the transwell migration assay (Figure 5G). Analysis of the expression of EMT marker genes E-cadherin, N-cadherin in mRNA by qRT-PCR and western blot also supported the above results (Figure 5I, 5J). Meanwhile, we also performed the transwell invasion assays in the loss-of-function and gain-of-function of *Epist* in KYSE30 cells (Materials and Methods). The invaded results were similar to those of transwell migration assays (Figure 5D-5G).

Additionally, it is well-established that TGF-β could promote cancer metastasis through its efforts on the tumor microenvironment [29]. Accordingly, we observed that the expression level of *Epist* was also decreased when the cells was treated by TGF-β (GSE54797) [30] (Figure 5H).

As a third line of investigation, the MTT assay was applied to measure the growth rate of KYSE30 cells over-expressing *Epist.* The results showed that over-expressing cell had a lower growth rate than controls (Figure 5K), indicating that *Epist* inhibited ESCC cell proliferation.

### *Epist* functions as a tumor suppressing lncRNA in ESCC

**Figure 6:**
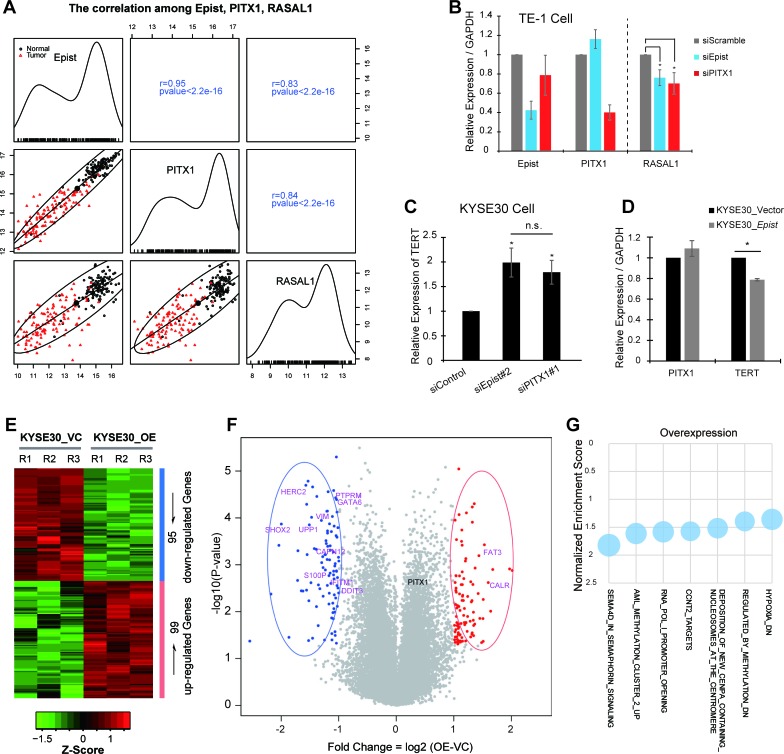
*Epist* acts as a tumor suppressor during ESCC tumorigenesis **A.** Scatter plot showing the expressional relationship among *Epist,* PITX1 and RASAL1. RASAL1 is one of the downstream targets of PITX1. The upper right squares show the Pearson correlation values between each other, and lower left squares show the scatter plot matrix and fitted expressional trend lines for the same comparisons. The black circle and the red triangle represent the expression score in tumor tissues and matched normal tissues, respectively. The diagonal shows the expression distribution across the 119 ESCC paired tissues. **B.**, **C.** siRNA-mediated loss-of-function of *Epist* results in the expression alteration of PITX1-target genes, RASAL1 in TE-1 cell **B.** and TERT in KYSE30 cell **C.**. **D.** KYSE30 stably overexpressing *Epist* cell line shows significantly reduced expression level of TERT than control. **E.** Heatmap of the differentially expressed genes revealed by microarray-based analysis between the KYSE30 cells overexpressing *Epist* and control RNAs, including 95 and 99 down- and up-regulated genes, respectively. **F.** The volcano plot shows the expressional alterations of all annotated genes. The red and blue points represent the up- and down-regulated genes, respectively, and corresponding ovals enclosed several previously identified cancer-associated genes (Up-regulated: FAT3, CALR Down-regulated: VIM, GATA6, DDIT3, HERC2, UPP1, S100P, IFITM1, SHOX2, PTPRM, CAPN12). **G.** The selected genesets from the GSEA analysis. The y axis represents the normalized enrichment score and the relative circular area represents the significant level (−log10(Nominal *p*-value)).

Next, to address how *Epist* inhibits the ESCC tumorigenesis, we firstly examined the expression between *Epist* and PITX1 target genes. According to the previous genetic screening report, PITX1 suppresses tumorigenicity by down-regulating the RAS pathway (oncogenic signaling pathway) through RASAL1 [31]. Thus, we calculated the correlation between the expression levels of RASAL1 and *Epist* in the cohort of 119 ESCC patients. Consequently, we observed that *Epist* expression positively and significantly correlated with RASAL1 (Figure 6A). Furthermore, siRNA-mediated knock-down of either PITX1 or *Epist* produced similar effects on the expression level of RASAL1 in TE-1 cell line (Figure 6B). PITX1 has also been identified as a suppressor of telomerase reverse transcriptase (TERT) in tumorigenesis [32]. Up-regulation of TERT is a key component of the transformation processes in many malignant cancer cells. We therefore examined the expression of TERT in KYSE30 cells after knock-down or over-expression of *Epist* and found they led to up-regulation and down-regulation of TERT level, respectively (Figure 6C-6D).

Besides, we performed the microarray analysis to determine which genes' expression was altered by overexpressing *Epist* through comparing the gene expression profile between KYSE30-overexpressing-*Epist* cells and controls, thus identified 95 and 99 down-regulated and up-regulated genes, respectively (Figure 6E). Expression levels of several previously reported genes implicated in cancer development and progression were altered, such as DDIT3, GATA6, UPP1, FAT3 (Figure 6F). These genes are involved in tumorigenesis through diverse mechanisms. In addition, the GSEA results also revealed plenty of biological processes were changed upon Epist overexpression (Figure 6G).

Collectively, all these results indicated that *Epist* acts as a tumor suppressor in ESCC tumorigenesis.

### Search for novel lincRNA transcripts and gene fusions in ESCC

In contrast to microarrays, RNA-seq can be employed to identify the novel transcripts across the genome. We used the single-end RNA-seq data (GSE29968) to identify novel lincRNA candidates according to the pipeline described in Materials and Methods (Figure S1B), and identified 31 novel lincRNA candidates (Table S7). The expression profile of these novel lincRNAs across the 3 ESCC paired tissues are given in Table S7.

Also, RNA-seq data can be used to find the gene fusions and has already led to the discovery of several gene fusions in some cancers [33], such as the *BCR-ABL1* fusion in K562 cell line. We employed the above RNA-seq data to detect expressed gene fusions. However, no recurrent gene fusions were identified in these data. The depth of the RNA-seq data we analyzed is comparable to that in a work identifying recurrent rearrangements of *CIITA* in Hodgkin lymphoma cell lines [34]. On the other hand, gene fusions were neither detected in an analysis on the Sézary syndrome [35]. Although we do not observe the gene fusions in ESCC, we still cannot rule out the possibilities that the fusion transcripts exist, such as the heterologous gene linking to an oncogene.

## DISCUSSION

The human genome encodes a large number of lncRNAs that are dynamically expressed across different developmental stages, that show highly tissue-, differentiation- and lineage-specific expression patterns, and that are implicated in diverse biological processes [36]. To date, a considerable number of lncRNAs have been identified and demonstrated to play important roles in many human cancers [17, 19]. In addition, the progress of high-throughput technology in recent years, such as microarray and RNA-seq, has made it much easier to profile the noncoding transcriptome than before.

### LncRNAs are extensively dysregulated in ESCC tumorigenesis

The work presented here investigated the differences in the long noncoding transcriptome profile between the esophageal squamous cell carcinoma and adjacent normal tissue using both microarray and deep RNA-seq data. The study identified several hundred ESCC-associated lncRNAs (ESCALs), the majority of which had previously not been reported to implicate in tumor progression.

Although a large number of lncRNAs have shown to be involved in diverse biological pathways, the molecular mechanisms by which most lncRNAs act are still not clearly understood. One of the emerging themes of lncRNA is controlling of gene expression. There are a number of lncRNAs reported to play gene regulation roles through recruiting chromatin modifying complexes [37], such as *HOTAIR* and the PRC2 complex [20] and *HOTTIP* and the WDR5/MLL complex [38]. Some lncRNAs are also function through guiding transcription factors to their downstream targets, such as *Paupar* with PAX6 [39] and *THRIL* with hnRNP-L [40]. In ESCC cancer cell lines, *Epist* functions as a tumor suppressor via affecting the expression levels of a number of cancer-associated genes (Figure 6E, 6F). Although *Epist* apparently does not affect the expression of PITX1, we have shown that *Epist* may regulate the expression levels of at least two genes (RASAL1, TERT), targets of PITX1. However, we observed the facts that *Epist* and PITX1 are coordinately activated, perhaps they are co-located and share the upstream regulatory elements, thus co-expressed (Figure 6A). However, we did not detect physical interaction between Epist and PITX1 (data not shown). The detailed molecular mechanisms of *Epist* exerts are still needed to further investigate.

There are several ESCALs showing fold difference greater than +/−20. Of which, *CASC9* (Homo sapiens cancer susceptibility candidate 9, LINC00981) has been recently validated in other cohorts of ESCC patients and reported to play the oncogenic roles in ESCC [41]. *LOC645638* is another example of ESCAL that was detected down-regulated markedly in all three datasets (~5 fold). Moreover, this lncRNA was significantly associated with the ESCC patient survival (Figure 2D) and, like *Epist,* was also inferred to have biological relevance to metastasis and cell cycle processes (Table S6). A recent report has shown that *LOC645638* (lnc-DC), which is exclusively expressed in human conventional dendritic cells, promotes the phosphorylation of STAT3 Y705 by preventing STAT3 binding to and dephosphorylation by SHP1 [42]. Future studies should be addressed whether this lncRNA has tumor suppressive activity, and if so, whether these mechanisms are important in this aspect.

### Inferring the biological functions of lncRNAs with paired tissues data

One of the major challenges concerning the study of lncRNAs is to determine their biological functions. Here we used the GSEA tool to predict the biological roles for the lncRNAs in ESCC paired tissues data through the “guilt-by-association” method [9]. For this purpose, the fold difference in expression (ΔE = T - N) in tissues (and not the expression value itself) was assigned to each probed lncRNA and protein-coding gene. Because the genomic landscape in ESCC was similar to that in head and neck squamous cell carcinomas (HNSCC), two metastatic gene signatures applied to HNSCC were used [43]. The correlation between lncRNAs and mRNAs was computed based on the fold difference values. The fold difference may reflect transcriptional responses in the gene regulatory network. Aberrantly expressed *HOTAIR* previously reported to promote metastasis, was taken as a positive control [20]. Our work here is the first report to establish the utility of paired tissues data to infer functional roles of lncRNA in cancerous pathways.

### *Epist* may play critical roles in development and differentiation

Recent efforts have shown that lncRNAs have higher tissue-specificity and less conservation than those of protein-coding genes [36]. In accordance with this notion, *Epist* is highly expressed in the esophagus (and to some extent in the prostate and colon). *Epist* is also not appreciably conserved in vertebrates, although it appears to be conserved among primates, indicating of a more recent evolution (Figure S7).

Besides, the GSEA results suggested that among the genes, which were positively correlated with *Epist* in the microarray data, there were significantly enriched for genes belonging to the epithelial differentiation module (Figure S6B). Also, the bivalent domain (a chromatin region marked by both the active mark, H3K4me3 and the repressive mark, H3K27me3) [44] occurs at the promoter region of *Epist* and PITX1 in the H1-hESC cell line (ENCODE ChIP-seq data) (Figure S6C). Bivalent domains were proposed to represent a state in which the developmental and differential genes in ES cells are silent while at the same time being kept poised for activation [44]. Previously, lncRNA *Fendrr*, which controls the cell fate of lateral mesoderm [16] and in lung development [13], also possesses a bivalent domain in ES cells, and available ChromHMM data also showed signs of the poised promoter state at the *Epist* locus in diverse cell types. PITX1 has been reported to plays critical roles in specifying hindlimb morphology [45], thus, taken together, we may further postulate that bivalent chromatin marks *Epist* having essential roles in development and differentiation similar to those of PITX1.

Further investigation should address the functional significance of other ESCALs in order to elucidate the detailed mechanisms by which they exert. The application of recently developed technologies to capture the chromatin binding profile of ncRNAs on a genome scale, such as chromatin isolation by RNA purification (ChIRP) [46] and RNA antisense purification (RAP) [47], will probably contribute to unveil the detailed molecular mechanisms of how lncRNAs control gene expression and chromatin state.

This study on a Chinese patient group has yielded new insights into the pathogenesis of ESCC, providing evidence that long noncoding transcripts may be regulators of tumorigenesis. Further work on the detailed mechanism by which lncRNAs act will be important for understanding how these transcripts may affect disease or cancer states and could provide key insights into therapeutic targeting.

## MATERIALS AND METHODS

### Microarray and RNA-seq data analysis

The transcriptome profiles for 119 pairs of ESCCs and patient-matched normal tissues, were measured by the combined mRNA + lncRNA V2.0 microarray on an Agilent platform. The microarray data had previously been deposited on the Gene Expression Omnibus, under accession number GSE53625. Data processing was similar to our previous work [22]. Briefly, for the noncoding RNA probes, we established a probe map for mapping the probe sequences uniquely to the long noncoding RNA collections (GENCODE v19, UCSC NR* transcript and Cabili M. N. *et al.* [23]). Median values were used for multiple probes corresponding to the same noncoding gene. Quantile normalization, and imputation for missing values using a KNN-based method were processed in R. For calling of differentially expressed genes, the fold differences (>=2), FDR (<0.001) and average expression were used (the probed transcripts were discarded, if both normal and tumor values were less than the average value across the samples. The average expression value is 6.799 for lncRNA and 9.963 for protein-coding gene, shown as the dash lines in Figure 1A). For the Gene Ontology enrichment analysis, the DAVID webserver [48] was used.

The methods calling of differentially expressed lncRNAs for RNA-seq data were documented in Document S1. We computed the FPKM value as the expression level. The detailed summary of the high-throughput data is listed in Figure S2. Genome coverage wiggle files were generated with BEDTools [49]. Additional RNA-seq data from human tissues, cell types and cancer cell types were retrieved from the Illumina Human Body Map Project [23] and the human ENCODE project [50]. To identify the novel lincRNA transcripts, single-exon transcripts were eliminated from the Cufflinks-assembled transcriptome [23]. De novo transcriptome assembly was processed by the Trinity software [51].

### ESCC cell lines and culture conditions

The ESCC cell lines (KYSE510, KYSE450, KYSE150, KYSE30, EC109 and TE-1) were purchased from Cell Resources Center, IBMS, CAMS/PUMC. The immortalized normal esophageal epithelial cell line Het-1A was purchased from ATCC, not authenticated before use. The STR profile of KYSE30 cell line was authenticated at GENEWIZ, Beijing. The TE-1 and EC109 cell lines were authenticated by Cell Resources Center, were maintained using standard media and conditions. Specifically, KYSE510, KYSE450, KYSE150, KYSE30, EC109 and TE-1 cells were maintained in RPMI 1640 (Life Technology) supplemented with 10% FBS. Het-1A was maintained in BEBM (Lonza). The flasks used to culture Het-1A cells were pre-coated with a mixture of 0.01 mg/mL fibronectin, 0.03 mg/mL bovine collagen type I and 0.01 mg/mL bovine serum albumin dissolved in culture medium. KYSE30 stably overexpressing *Epist* or control vector cell lines were generated by transfected lentiviral constructs encoding *Epist* or blank RNA for 48 h. GFP-positive cells were selected on 2 μg/ml puromycin for one week.

### Transcriptome-wide analysis of KYSE30 cell line overexpressing *Epist*

Total RNAs were isolated from the KYSE30 cells overexpressing *Epist* or control vector using the Trizol (three biological replicates), followed by DNaseI treatment. The quality of RNAs were assessed with Nanodrop 2000. 500 ng RNAs were used to generate fluorescence-labelled cDNA for hybridization according to the Agilent standard workflow. The microarray hybridization experiment was performed at the CapitalBio Corporation, Beijing. Arrays were scanned with the Agilent DNA Microarray Scanner and processed with the Agilent Feature Extraction v10.7, then quantile normalized using the R limma package. The fold change and two-tailed Student's *t* test were applied for statistical analysis of differentially expressed genes. Heatmap and Scatterplot were drawn with the functions in R. The microarray data have been deposited in the GEO database under accession number GSE64792.

### Rapid amplification of cDNA ends (RACE), northern blot and quantitative RT-PCR (qRT-PCR)

Total RNAs were isolated from the cell lines (Het-1A, EC109, KYSE510, KYSE450, TE-1, KYSE30, KYSE150) with the Trizol reagent (Invitrogen) according to the manufacturer's instructions, and then subjected to DNaseI treatment. 3′- and 5′-RACE were performed using the RLM RACE kit (Ambion AM1700) and 5′-RACE System for Rapid Amplification cDNA Ends (Life Technology, 18374-058).

For the qRT-PCRs, the SuperScript III reverse transcriptase was employed to synthetize the first-strand cDNA. qPCR analysis was performed with 0.5 μL cDNA as template in a 20 μL reaction volumes using SYBR green master mixture on the Rotor-Gene^®^ Q real-time cycler (Qiagen). The expression levels were normalized to GAPDH and the relative expression was calculated by the delta-delta Ct method. All analyses shown were carried out on at least three biological replicates for each sample; *P-values* were calculated by Student's *t-test* from the biological replicates.

The Northern blot was performed as described [52]. All primers used are listed in the Table S8.

### Nuclear localization

The nuclear and cytoplasmic fractions of TE-1 cells were isolated with the NE-PER Nuclear and Cytoplasmic Extraction Reagents (Thermo). The total RNA isolated from the subcellular fractions was assessed by qRT-PCR. GAPDH and ACTB serve as the cytosolic controls, and U1 as the nuclear control.

### Survival analysis with probed genes for ESCC

For each probe in the microarray assay, we performed in parallel survival analysis with the expression value (T) of tumor tissues and the difference in expression value between tumor and adjacent normal tissues (ΔE = T - N). We labelled the tumor expression or expression difference of each probe as “high” or “low” according to whether the value was higher or lower than the average of T or ΔE, respectively, across the 119 pairs of ESCC samples. The log-rank test was used to measure whether the survival time was significantly different between the “high” and “low” expression of patient groups, the probe was included for further consideration if p-values for both T and ΔE were less than 0.05. The Kaplan-Meier plots were drawn using the R packages. Clinical information concerning the patients is found in Li *et al*. [22].

### Gene set enrichment analysis

For the GSEA [53], fold difference (ΔE = T - N) of every probe of ESCALs in the microarray across the 119 paired samples was considered as a numeric vector, and all protein-coding genes were ranked by the Pearson metric with our python script. Genes whose correlation was measured by multiple probes were consolidated using the maximum values obtained with these probes. We employed the GseaPreRanked tool to identify the enriched gene-set collections in c2.all.v4.0.symbols.gmt of MSigDB with a permutation of 1000. The threshold for the nominal p-value and FDR q-value were set to 0.01 and 0.05, respectively. For calculating the correlation with metastasis, the three paired gene sets from the MSigDB Chemical and Genetic Perturbations (CGP) categories were taken to represent metastasis gene signature (JAEGER_METASTASIS_UP and _DN [54], RICKMAN_METASTASIS_UP and _DN [55], CROMER_METASTASIS_UP and _DN [56]). The KEGG_CELL_CYCLE and the KEGG_APOPTOSIS get sets were used to assess the associations with cell cycle and apoptosis, respectively.

### Lentivirus production and purification

The *Epist* sequence was cloned into the lentivector pCDH-MSCV-MCS-EF1-GFP+Puro cDNA Cloning and Expression Vector (SBI CD713B-1). 293T cells were cultured in T-175 flasks at 40%~50% confluence before transfection. Transfection was performed using Lipofectamine 2000 (Life Technologies). For each flask, 20.25 μg of lentivectors, 13.2 μg of pMDL-g/p, 5.1 μg of RSV-REV and 7.125 μg of VSVG were added to 4 ml OptiMEM (Life Technologies). 100 μl of Lipofectamine 2000 was diluted in 4 ml OptiMEM and it was added to the plasmid mixture after 5 min. The complete mixture was incubated at room temperature for 20 min before being added to cells. After 6 h, the medium was changed to 30 ml DMEM + 20% FBS. The medium was collected at 48 h and 72 h and centrifuged at 3,000 rpm at 4 °C for 10 min to pellet the cell debris. The supernatant was filtered through a 0.45 μm low protein binding membrane. The virus was ultracentrifuged at 20000 g for 2 h at 4°C and then resuspended overnight at 4°C in PBS. Aliquots were stored at −80°C.

### Transwell migration assay

For knock-down of *Epist*, KYSE30 cells were transfected with siRNA, trypsinized after 24 h, and counted with a Coulter counter. For overexpression of *Epist*, KYSE30 cells stably expressing *Epist* or the control vector were trypsinized, and counted with a Coulter counter. 35,000 cells were seeded in the upper chamber of Transwell (Corning 3422) with no serum medium, while 10% FBS medium was added to the lower chamber as a chemoattractant. After 24 h incubation at 37°C, 5% CO_2_ atmosphere, the non-migrating cells were gently removed with a cotton swab. Migrated cells located on the lower side of the chamber were stained with crystal violet, air dried and photographed. Migrated cells were eluted with 10% acetic acid, and absorbance measured at 560 nm using spectrophotometer.

### Transwell invasion assay

We diluted thawed BD Matrigel matrix (BD 356230) into coating buffer (0.01M Tris pH 8.0, 0.7% NaCl) to a final concentration of 250 μg/ml, mix gently and thoroughly, followed by adding 0.1 ml of the diluted Matrigel matrix coating solution into the upper chamber of the Transwell (Corning 3422) and incubating the coated Transwell plate at 37°C for 2 h. Any pipets, syringes, or containers that will come in contact with BD Matrigel matrix must be chilled prior to use. Cultured 100,000 cells in the coated upper chamber, the rest operation is same as Transwell migration assay mentioned above.

All the images of Transwell migration and invasion assays were captured using an Olympus microscope imaging systems.

## SUPPLEMENTARY MATERIALS FIGURES AND TABLES





















## References

[1] Jemal A, Bray F, Center MM, Ferlay J, Ward E, Forman D (2011). Global cancer statistics. CA: a cancer journal for clinicians.

[2] Yang L, Parkin DM, Ferlay J, Li L, Chen Y (2005). Estimates of cancer incidence in China for 2000 and projections for 2005. Cancer epidemiology, biomarkers & prevention : a publication of the American Association for Cancer Research, cosponsored by the American Society of Preventive Oncology.

[3] Lima SC, Hernandez-Vargas H, Simao T, Durand G, Kruel CD, Le Calvez-Kelm F, Ribeiro Pinto LF, Herceg Z (2011). Identification of a DNA methylome signature of esophageal squamous cell carcinoma and potential epigenetic biomarkers. Epigenetics : official journal of the DNA Methylation Society.

[4] Li JS, Ying JM, Wang XW, Wang ZH, Tao Q, Li LL (2013). Promoter methylation of tumor suppressor genes in esophageal squamous cell carcinoma. Chin J Cancer.

[5] Ma S, Bao JYJ, Kwan PS, Chan YP, Tong CM, Fu L, Zhang N, Tong AHY, Qin YR, Tsao SW, Chan KW, Lok S, Guan XY (2012). Identification of PTK6, via RNA Sequencing Analysis, as a Suppressor of Esophageal Squamous Cell Carcinoma. Gastroenterology.

[6] Tong M, Chan KW, Bao JY, Wong KY, Chen JN, Kwan PS, Tang KH, Fu L, Qin YR, Lok S, Guan XY, Ma S (2012). Rab25 is a tumor suppressor gene with antiangiogenic and anti-invasive activities in esophageal squamous cell carcinoma. Cancer Res.

[7] Ding DP, Chen ZL, Zhao XH, Wang JW, Sun J, Wang Z, Tan FW, Tan XG, Li BZ, Zhou F, Shao K, Li N, Qiu B, He J (2011). miR-29c induces cell cycle arrest in esophageal squamous cell carcinoma by modulating cyclin E expression. Carcinogenesis.

[8] Xu X, Chen Z, Zhao X, Wang J, Ding D, Wang Z, Tan F, Tan X, Zhou F, Sun J, Sun N, Gao Y, Shao K, Li N, Qiu B, He J (2012). MicroRNA-25 promotes cell migration and invasion in esophageal squamous cell carcinoma. Biochem Biophys Res Commun.

[9] Guttman M, Amit I, Garber M, French C, Lin MF, Feldser D, Huarte M, Zuk O, Carey BW, Cassady JP, Cabili MN, Jaenisch R, Mikkelsen TS, Jacks T, Hacohen N, Bernstein BE (2009). Chromatin signature reveals over a thousand highly conserved large non-coding RNAs in mammals. Nature.

[10] Klattenhoff CA, Scheuermann JC, Surface LE, Bradley RK, Fields PA, Steinhauser ML, Ding H, Butty VL, Torrey L, Haas S, Abo R, Tabebordbar M, Lee RT, Burge CB, Boyer LA (2013). Braveheart, a long noncoding RNA required for cardiovascular lineage commitment. Cell.

[11] Ulitsky I, Shkumatava A, Jan CH, Sive H, Bartel DP (2011). Conserved function of lincRNAs in vertebrate embryonic development despite rapid sequence evolution. Cell.

[12] Guttman M, Donaghey J, Carey BW, Garber M, Grenier JK, Munson G, Young G, Lucas AB, Ach R, Bruhn L, Yang X, Amit I, Meissner A, Regev A, Rinn JL, Root DE (2011). lincRNAs act in the circuitry controlling pluripotency and differentiation. Nature.

[13] Sauvageau M, Goff LA, Lodato S, Bonev B, Groff AF, Gerhardinger C, Sanchez-Gomez DB, Hacisuleyman E, Li E, Spence M, Liapis SC, Mallard W, Morse M, Swerdel MR, D'Ecclessis MF, Moore JC (2013). Multiple knockout mouse models reveal lincRNAs are required for life and brain development. eLife.

[14] Rinn JL, Kertesz M, Wang JK, Squazzo SL, Xu X, Brugmann SA, Goodnough LH, Helms JA, Farnham PJ, Segal E, Chang HY (2007). Functional demarcation of active and silent chromatin domains in human HOX loci by noncoding RNAs. Cell.

[15] Kotake Y, Nakagawa T, Kitagawa K, Suzuki S, Liu N, Kitagawa M, Xiong Y (2011). Long non-coding RNA ANRIL is required for the PRC2 recruitment to and silencing of p15(INK4B) tumor suppressor gene. Oncogene.

[16] Grote P, Wittler L, Hendrix D, Koch F, Wahrisch S, Beisaw A, Macura K, Blass G, Kellis M, Werber M, Herrmann BG (2013). The Tissue-Specific lncRNA Fendrr Is an Essential Regulator of Heart and Body Wall Development in the Mouse. Developmental cell.

[17] Prensner JR, Iyer MK, Balbin OA, Dhanasekaran SM, Cao Q, Brenner JC, Laxman B, Asangani IA, Grasso CS, Kominsky HD, Cao X, Jing X, Wang X, Siddiqui J, Wei JT, Robinson D (2011). Transcriptome sequencing across a prostate cancer cohort identifies PCAT-1, an unannotated lincRNA implicated in disease progression. Nat Biotechnol.

[18] Prensner JR, Iyer MK, Sahu A, Asangani IA, Cao Q, Patel L, Vergara IA, Davicioni E, Erho N, Ghadessi M, Jenkins RB, Triche TJ, Malik R, Bedenis R, McGregor N, Ma T (2013). The long noncoding RNA SChLAP1 promotes aggressive prostate cancer and antagonizes the SWI/SNF complex. Nat Genet.

[19] Brunner AL, Beck AH, Edris B, Sweeney RT, Zhu SX, Li R, Montgomery K, Varma S, Gilks T, Guo X, Foley JW, Witten DM, Giacomini CP, Flynn RA, Pollack JR, Tibshirani R (2012). Transcriptional profiling of lncRNAs and novel transcribed regions across a diverse panel of archived human cancers. Genome Biol.

[20] Gupta RA, Shah N, Wang KC, Kim J, Horlings HM, Wong DJ, Tsai MC, Hung T, Argani P, Rinn JL, Wang Y, Brzoska P, Kong B, Li R, West RB, van de Vijver MJ (2010). Long non-coding RNA HOTAIR reprograms chromatin state to promote cancer metastasis. Nature.

[21] Kogo R, Shimamura T, Mimori K, Kawahara K, Imoto S, Sudo T, Tanaka F, Shibata K, Suzuki A, Komune S, Miyano S, Mori M (2011). Long noncoding RNA HOTAIR regulates polycomb-dependent chromatin modification and is associated with poor prognosis in colorectal cancers. Cancer Res.

[22] Li J, Chen Z, Tian L, Zhou C, He MY, Gao Y, Wang S, Zhou F, Shi S, Feng X, Sun N, Liu Z, Skogerboe G, Dong J, Yao R, Zhao Y (2014). LncRNA profile study reveals a three-lncRNA signature associated with the survival of patients with oesophageal squamous cell carcinoma. Gut.

[23] Cabili MN, Trapnell C, Goff L, Koziol M, Tazon-Vega B, Regev A, Rinn JL (2011). Integrative annotation of human large intergenic noncoding RNAs reveals global properties and specific subclasses. Genes Dev.

[24] Tano K, Mizuno R, Okada T, Rakwal R, Shibato J, Masuo Y, Ijiri K, Akimitsu N (2010). MALAT-1 enhances cell motility of lung adenocarcinoma cells by influencing the expression of motility-related genes. Febs Lett.

[25] Chen G, Wang Z, Wang D, Qiu C, Liu M, Chen X, Zhang Q, Yan G, Cui Q (2013). LncRNADisease: a database for long-non-coding RNA-associated diseases. Nucleic Acids Res.

[26] Li X, Wu Z, Mei Q, Guo M, Fu X, Han W (2013). Long non-coding RNA HOTAIR, a driver of malignancy, predicts negative prognosis and exhibits oncogenic activity in oesophageal squamous cell carcinoma. Br J Cancer.

[27] Kundaje A, Meuleman W, Ernst J, Bilenky M, Yen A, Heravi-Moussavi A, Kheradpour P, Zhang Z, Wang J, Ziller MJ, Amin V, Whitaker JW, Schultz MD, Ward LD, Sarkar A, Quon G (2015). Integrative analysis of 111 reference human epigenomes. Nature.

[28] Wang S, Zhan M, Yin J, Abraham JM, Mori Y, Sato F, Xu Y, Olaru A, Berki AT, Li H, Schulmann K, Kan T, Hamilton JP, Paun B, Yu MM, Jin Z (2006). Transcriptional profiling suggests that Barrett's metaplasia is an early intermediate stage in esophageal adenocarcinogenesis. Oncogene.

[29] Padua D, Massague J (2009). Roles of TGFβ in metastasis. Cell Res.

[30] Yuan JH, Yang F, Wang F, Ma JZ, Guo YJ, Tao QF, Liu F, Pan W, Wang TT, Zhou CC, Wang SB, Wang YZ, Yang Y, Yang N, Zhou WP, Yang GS (2014). A long noncoding RNA activated by TGF-β promotes the invasion-metastasis cascade in hepatocellular carcinoma. Cancer cell.

[31] Kolfschoten IG, van Leeuwen B, Berns K, Mullenders J, Beijersbergen RL, Bernards R, Voorhoeve PM, Agami R (2005). A genetic screen identifies PITX1 as a suppressor of RAS activity and tumorigenicity. Cell.

[32] Qi DL, Ohhira T, Fujisaki C, Inoue T, Ohta T, Osaki M, Ohshiro E, Seko T, Aoki S, Oshimura M, Kugoh H (2011). Identification of PITX1 as a TERT suppressor gene located on human chromosome 5. Mol Cell Biol.

[33] Maher CA, Kumar-Sinha C, Cao X, Kalyana-Sundaram S, Han B, Jing X, Sam L, Barrette T, Palanisamy N, Chinnaiyan AM (2009). Transcriptome sequencing to detect gene fusions in cancer. Nature.

[34] Steidl C, Shah SP, Woolcock BW, Rui L, Kawahara M, Farinha P, Johnson NA, Zhao Y, Telenius A, Neriah SB, McPherson A, Meissner B, Okoye UC, Diepstra A, van den Berg A, Sun M (2011). MHC class II transactivator CIITA is a recurrent gene fusion partner in lymphoid cancers. Nature.

[35] Lee CS, Ungewickell A, Bhaduri A, Qu K, Webster DE, Armstrong R, Weng WK, Aros CJ, Mah A, Chen RO, Lin M, Sundram U, Chang HY, Kretz M, Kim YH, Khavari PA (2012). Transcriptome sequencing in Sezary syndrome identifies Sezary cell and mycosis fungoides-associated lncRNAs and novel transcripts. Blood.

[36] Ulitsky I, Bartel DP (2013). lincRNAs: Genomics, Evolution, and Mechanisms. Cell.

[37] Khalil AM, Guttman M, Huarte M, Garber M, Raj A, Rivea Morales D, Thomas K, Presser A, Bernstein BE, van Oudenaarden A, Regev A, Lander ES, Rinn JL (2009). Many human large intergenic noncoding RNAs associate with chromatin-modifying complexes and affect gene expression. Proceedings of the National Academy of Sciences.

[38] Wang KC, Yang YW, Liu B, Sanyal A, Corces-Zimmerman R, Chen Y, Lajoie BR, Protacio A, Flynn RA, Gupta RA, Wysocka J, Lei M, Dekker J, Helms JA, Chang HY (2011). A long noncoding RNA maintains active chromatin to coordinate homeotic gene expression. Nature.

[39] Vance KW, Sansom SN, Lee S, Chalei V, Kong L, Cooper SE, Oliver PL, Ponting CP (2014). The long non-coding RNA Paupar regulates the expression of both local and distal genes. The EMBO journal.

[40] Li Z, Chao TC, Chang KY, Lin N, Patil VS, Shimizu C, Head SR, Burns JC, Rana TM (2014). The long noncoding RNA THRIL regulates TNFalpha expression through its interaction with hnRNPL. Proceedings of the National Academy of Sciences of the United States of America.

[41] Hao Y, Wu W, Shi F, Dalmolin RJ, Yan M, Tian F, Chen X, Chen G, Cao W (2015). Prediction of long noncoding RNA functions with co-expression network in esophageal squamous cell carcinoma. BMC Cancer.

[42] Wang P, Xue Y, Han Y, Lin L, Wu C, Xu S, Jiang Z, Xu J, Liu Q, Cao X (2014). The STAT3-binding long noncoding RNA lnc-DC controls human dendritic cell differentiation. Science.

[43] Song Y, Li L, Ou Y, Gao Z, Li E, Li X, Zhang W, Wang J, Xu L, Zhou Y, Ma X, Liu L, Zhao Z, Huang X, Fan J, Dong L (2014). Identification of genomic alterations in oesophageal squamous cell cancer. Nature.

[44] Bernstein BE, Mikkelsen TS, Xie X, Kamal M, Huebert DJ, Cuff J, Fry B, Meissner A, Wernig M, Plath K, Jaenisch R, Wagschal A, Feil R, Schreiber SL, Lander ES (2006). A bivalent chromatin structure marks key developmental genes in embryonic stem cells. Cell.

[45] Infante CR, Park S, Mihala AG, Kingsley DM, Menke DB (2013). Pitx1 broadly associates with limb enhancers and is enriched on hindlimb cis-regulatory elements. Dev Biol.

[46] Chu C, Qu K, Zhong FL, Artandi SE, Chang HY (2011). Genomic maps of long noncoding RNA occupancy reveal principles of RNA-chromatin interactions. Mol Cell.

[47] Engreitz JM, Pandya-Jones A, McDonel P, Shishkin A, Sirokman K, Surka C, Kadri S, Xing J, Goren A, Lander ES, Plath K, Guttman M (2013). The Xist lncRNA exploits three-dimensional genome architecture to spread across the X chromosome. Science.

[48] Huang da W, Sherman BT, Lempicki RA (2009). Systematic and integrative analysis of large gene lists using DAVID bioinformatics resources. Nat Protoc.

[49] Quinlan AR, Hall IM (2010). BEDTools: a flexible suite of utilities for comparing genomic features. Bioinformatics.

[50] Dunham I, Kundaje A, Aldred SF, Collins PJ, Davis CA, Doyle F, Epstein CB, Frietze S, Harrow J, Kaul R, Khatun J, Lajoie BR, Landt SG, Lee BK, Pauli F, Rosenbloom KR (2012). An integrated encyclopedia of DNA elements in the human genome. Nature.

[51] Haas BJ, Papanicolaou A, Yassour M, Grabherr M, Blood PD, Bowden J, Couger MB, Eccles D, Li B, Lieber M, Macmanes MD, Ott M, Orvis J, Pochet N, Strozzi F, Weeks N (2013). De novo transcript sequence reconstruction from RNA-seq using the Trinity platform for reference generation and analysis. Nat Protoc.

[52] Luo H, Sun Y, Wei G, Luo J, Yang X, Liu W, Chen R (2015). The Functional Characterization of Long Non-coding RNA Lnc_bc060912 in Human Lung Carcinoma Cells. Biochemistry-us.

[53] Subramanian A, Tamayo P, Mootha VK, Mukherjee S, Ebert BL, Gillette MA, Paulovich A, Pomeroy SL, Golub TR, Lander ES, Mesirov JP (2005). Gene set enrichment analysis: a knowledge-based approach for interpreting genome-wide expression profiles. Proceedings of the National Academy of Sciences of the United States of America.

[54] Jaeger J, Koczan D, Thiesen HJ, Ibrahim SM, Gross G, Spang R, Kunz M (2007). Gene expression signatures for tumor progression, tumor subtype, and tumor thickness in laser-microdissected melanoma tissues. Clin Cancer Res.

[55] Rickman DS, Millon R, De Reynies A, Thomas E, Wasylyk C, Muller D, Abecassis J, Wasylyk B (2008). Prediction of future metastasis and molecular characterization of head and neck squamous-cell carcinoma based on transcriptome and genome analysis by microarrays. Oncogene.

[56] Cromer A, Carles A, Millon R, Ganguli G, Chalmel F, Lemaire F, Young J, Dembele D, Thibault C, Muller D, Poch O, Abecassis J, Wasylyk B (2004). Identification of genes associated with tumorigenesis and metastatic potential of hypopharyngeal cancer by microarray analysis. Oncogene.

