# Supplementary material for: Transcriptome profiling of esophageal squamous cell carcinoma reveals a long noncoding RNA acting as a tumor suppressor
Source: Oncotarget. 2015 May 19;6(19):17065–80. doi: 10.18632/oncotarget.4185 (PMC4627292; doi:10.18632/oncotarget.4185)
Supplement: Supplementary file 1 [file oncotarget-06-17065-s001.pdf]

Transcriptome profiling of esophageal squamous cell carcinoma reveals a long noncoding RNA acting as a tumor suppressor

Supplementary Material

Supplementary Documents

1. Document S1 Methods used for calling differentially expressed long noncoding RNAs for RNA-Seq data of GSE29968 and GSE32424.

2. Document S2 Materials and methods for coding potential analysis.

3. Document S3 Search for novel transcripts and fusion transcripts

Supplementary Tables

1. Table S1 The re-annotated probes for long noncoding RNAs collections.

2. Table S2A The expression table of the mRNA in the combined microarray across the 119 ESCC patients.

3. Table S2B The expression table of the lncRNA in the combined microarray across the 119 ESCC patients.

4. Table S3 The RNA-Seq expression profile of all coding and noncoding genes.

5. Table S4 ESCALs shows enriched expression specific to esophagus or ESCC. (**a**) The ESCALs overlapped with 3SEQ peaks. (**b**) Overlapped 3SEQ peaks shows differential expression patterns across the assayed tumor samples. (**c**) ESCALs intersecting with LncRNADisease database.

6. Table S5 Survival analysis tables for differentially expressed lncRNAs in microarray.

7. Table S6 GSEA result and the result data for Figure 3c.

8. Table S7 The novel transcripts identified from the GSE29968 data. Three pairs of ESCC and adjacent normal tissues.

9. Table S8 Primers and oligos used in this work.

A

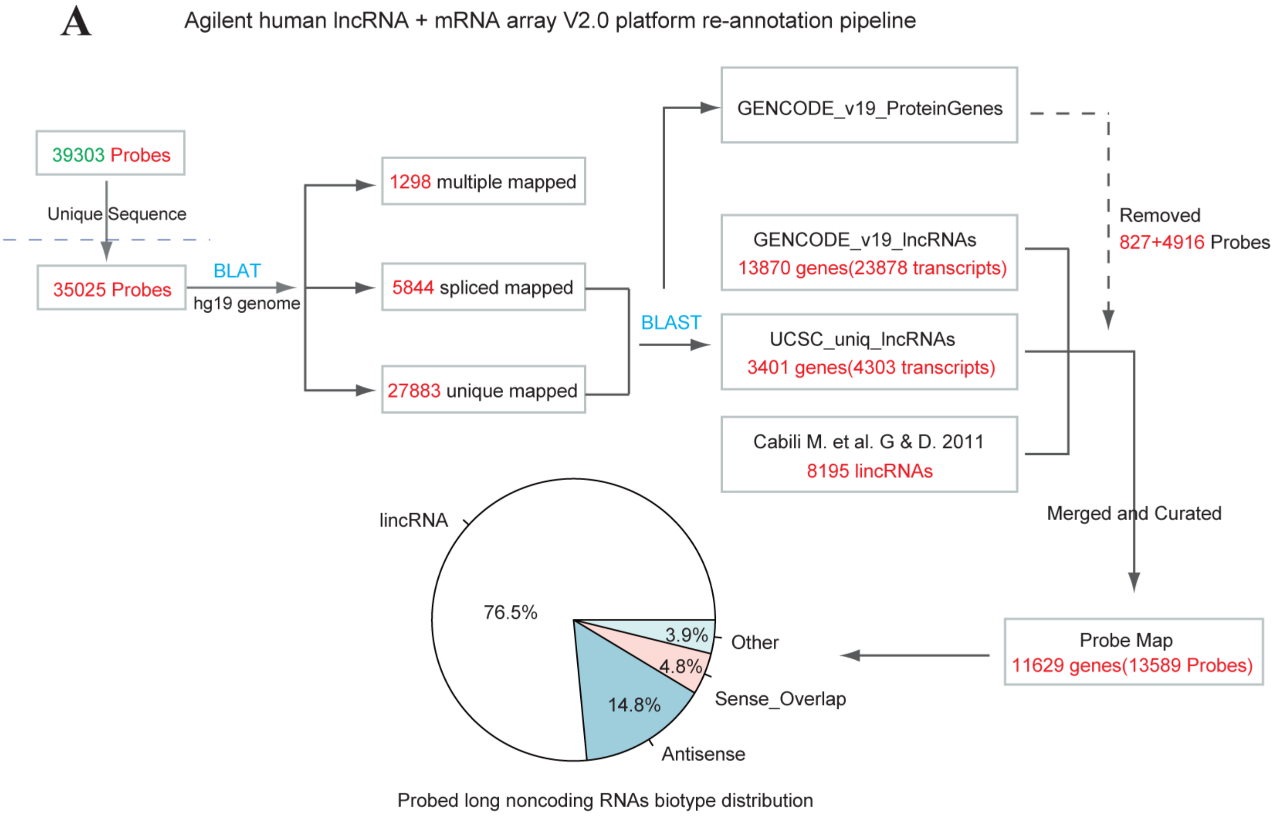

B

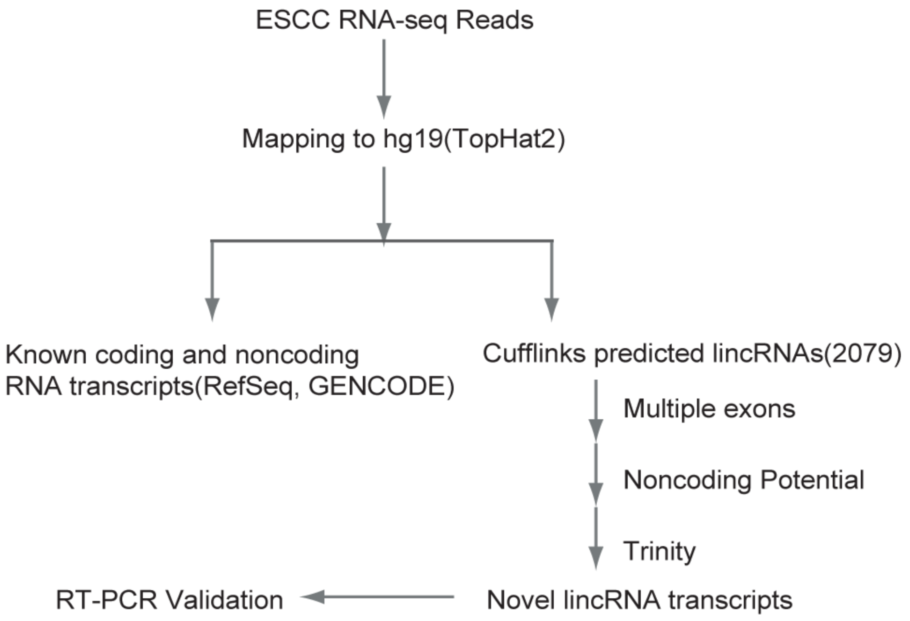

Figure S1: The pipeline for re-annotating the probes assayed in the combined microarray (**a**) and identifying the novel transcripts in RNA-Seq data (**b**).

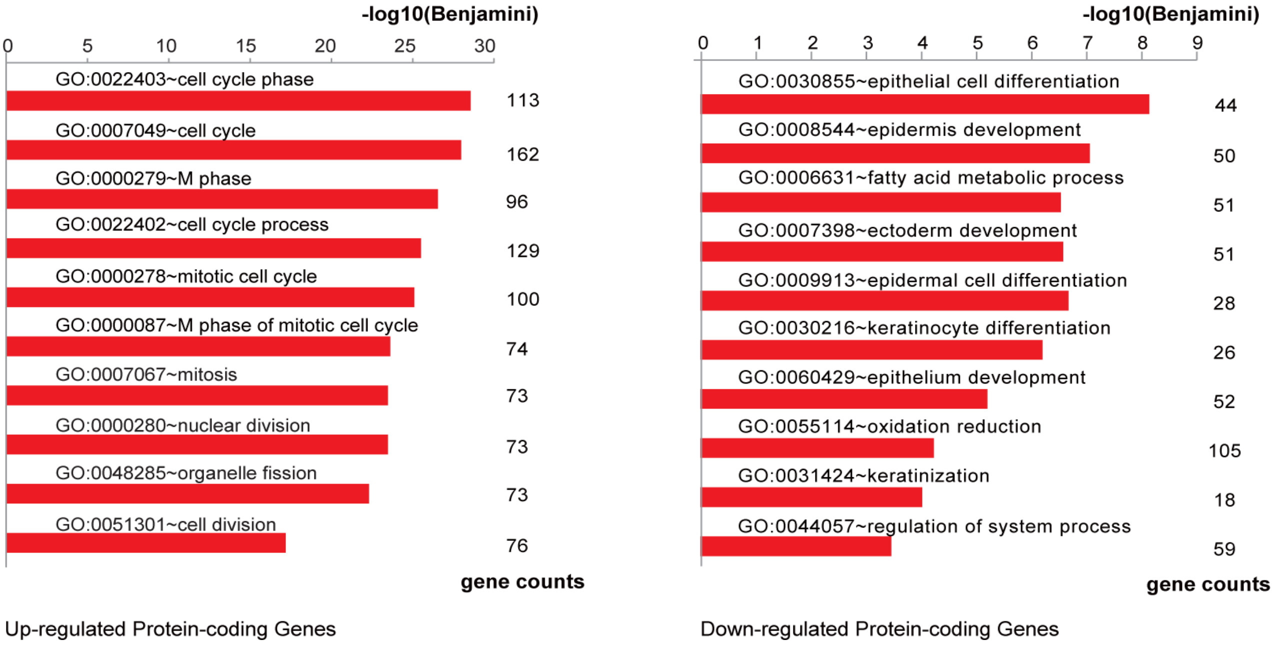

Figure S2: GO Enrichment analysis of up and down-regulated protein-coding genes between the tumor tissues and adjacent normal tissues across the 119 patients**.** A up-regulated genes. B down-regulated genes. The bar length denotes the -log10. Benjamini qValue. Top ten GO terms were shown.

A

Summary of the used high-throughput data

Data	Numbered	Methods	Up	Down
Microarray	N/A	Fold Change, t-test	292	537
GSE29968	16N,16T,18N,18T,19N,19T	Cuffnorm, custom	168	191
GSE32424	4N,5N,6N,8N,9N,1T,2T,3T,6T,7T,8T ,9T	SAM	193	249

Note:
The microarray and GSE29968 data measured the paired tissues, surrounding normal tissues and ESCC tumor samples. However, GSE32424 data measured unpaired tissues, 5 non-tumor and 7 ESCC samples.

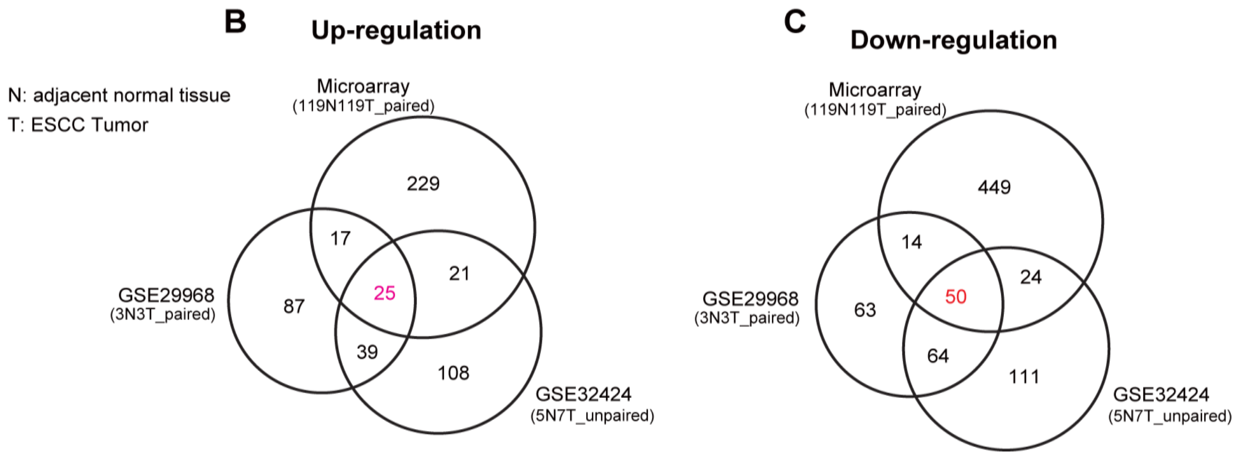

Figure S3: (**A**) the table of used high-throughput data as well as the differential analysis methods and the number of differential expressed lncRNAs in individual dataset. (**B,C**) the Venn diagram shows the overlap of differentially expressed long noncoding RNAs among these three datasets. (**B**) up-regulation. (**C**) Down-regulation.

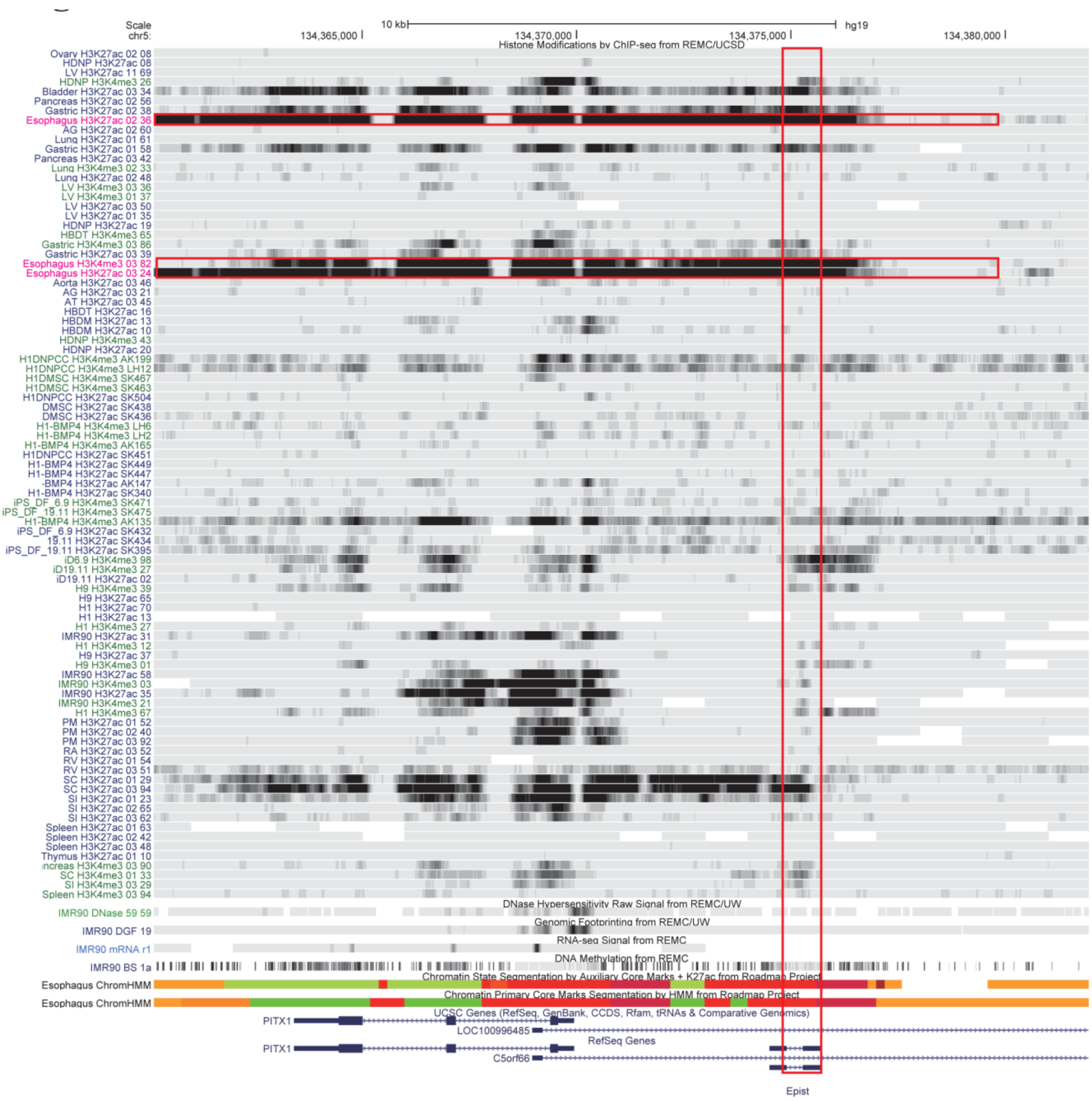

Figure S4: Epigenomic landscape of PITX1 and Epist locus.

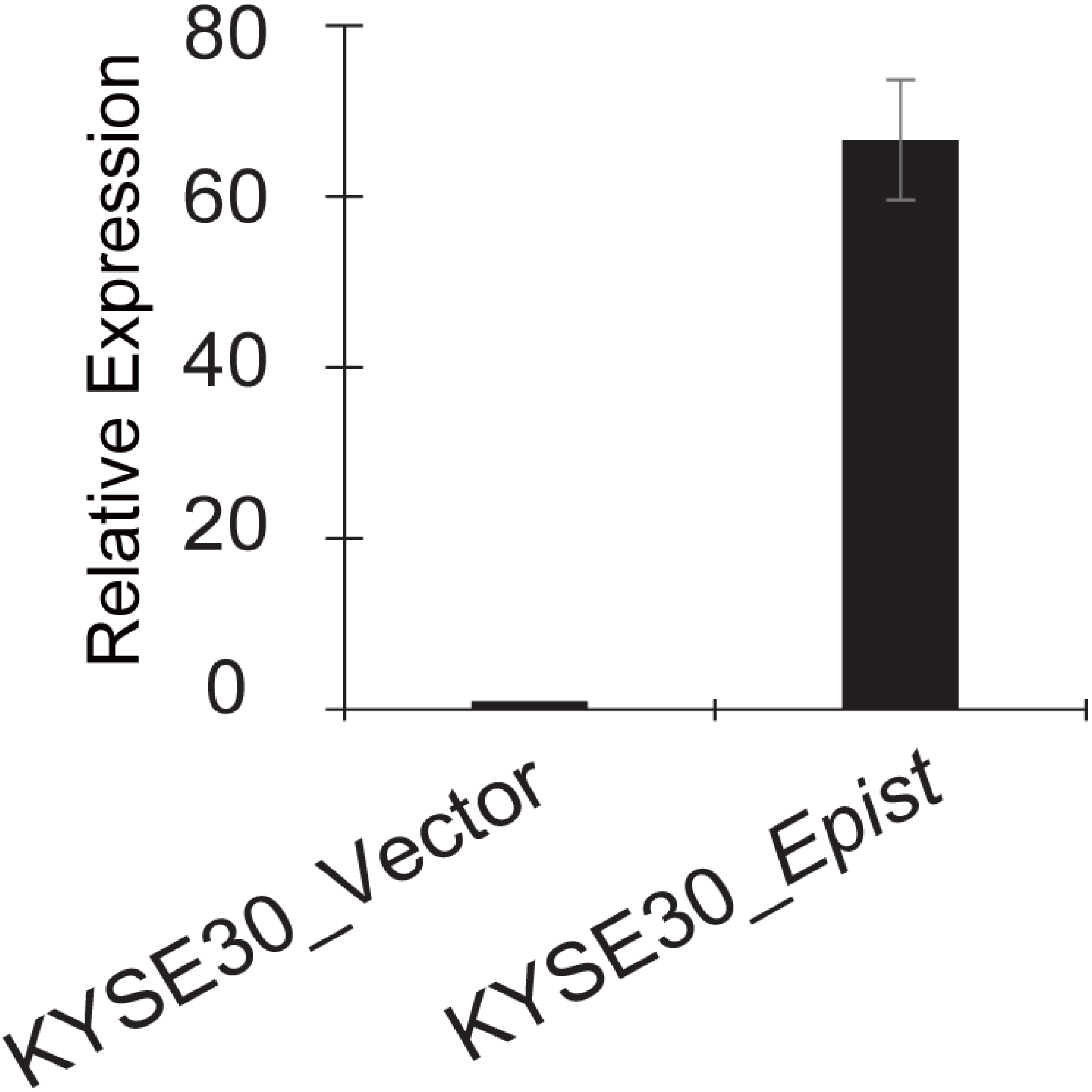

Figure S5: Expression levels of KYSE30 cells overexpressing the *Epist* or control vector sequence.

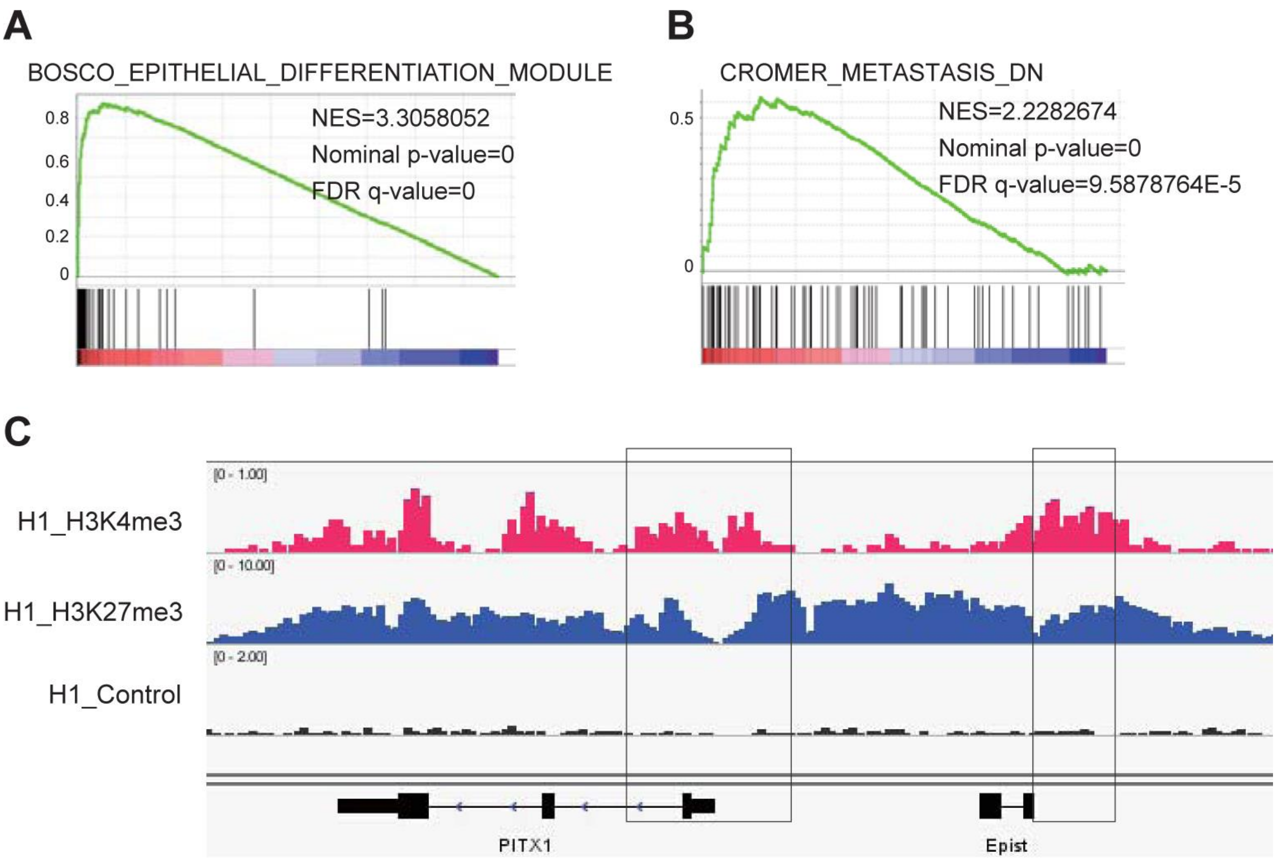

Figure S6: *Epist* is associated with metastasis and epithelial differentiation. (**A**) The positively correlated genes of *Epist* enriched the gene set of epithelial differentiation module from MSigDB. (**B**) *Epist*-correlated genes are enriched in the gene signatures of metastasis from *Cromer et al.* (**C**) The ChIP-Seq tracks for H3K4me3 and H3K27me3 in ENCODE cell line. hESC in PITX1-*Epist* locus. The gray boxes indicated the bivalent domains. the common peak region for both active and repress histone modifications.

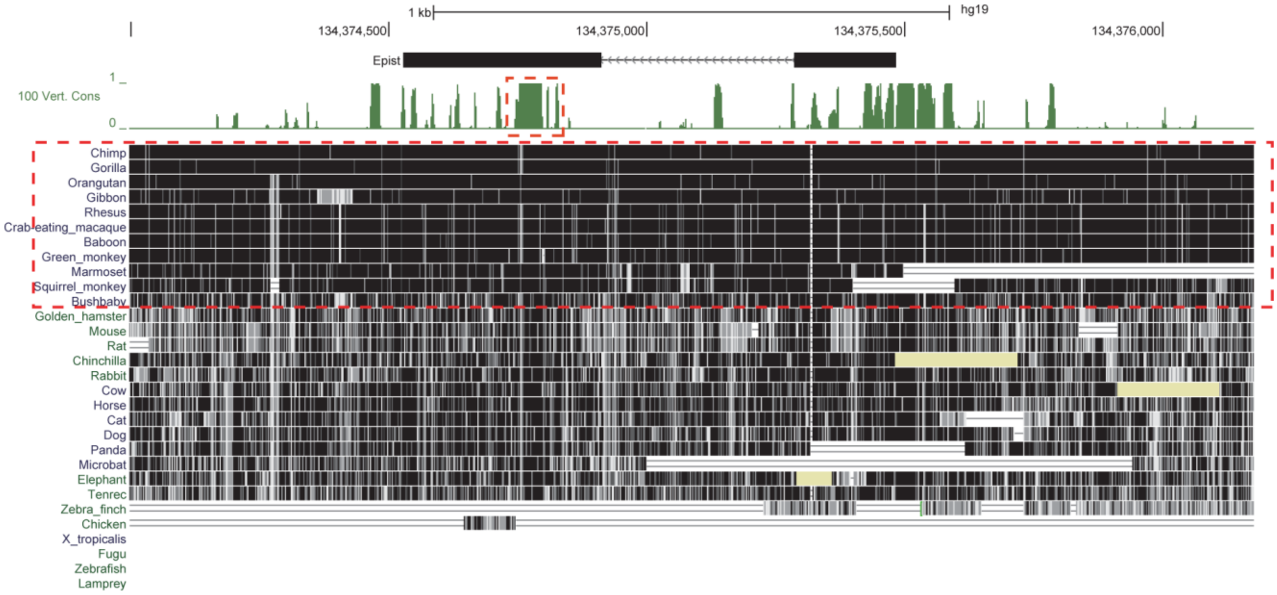

Figure S7: The conservation tracks for *Epist* locus. The lower red box suggests the species of primates. The upper red box indicates the conserved domain across the vertebrates.

Document S1: RNA-seq analysis for GSE29968 and GSE32424

The first RNA-Seq data were retrieved from GSE29968 [1], which sequenced the transcriptome of three pairs of ESCC and their matched adjacent normal tissue specimens (3N3T). The second RNA-Seq data, GSE32424 [2], sequenced 5 ESCC samples and 7 non-tumor samples (5N7T) with single-end reads. The GENCODE database[3] (v19) and UCSC genome browser annotations as well as the long intergenic noncoding RNAs from Cabili, M.N. *et al.* [4], were used as reference annotations. RNA-Seq reads were mapped to the human reference genome (hg19) using Tophat (v2.0.4) with the default parameters. For GSE29968, we measured the expression level of the transcripts with Cuffnorm program (v2.2.0) [5] by computing FPKM (Fragments Per Kilobase of transcript per Million mapped sequencing reads). Then, we removed the transcripts with the FPKM was lower than 1.0 across all the 3 ESCC paired tissues. Thereafter, the gene or lncRNA transcript was determined as differential expression when it has at least 2-fold change between all three pairs. For GSE32424, we used SAM [6] method to call the differentially expressed genes. The counts of gene sequencing tags is calculated by htseq-count program according to the common protocol based on the default parameters [7]. The detailed summary of the high-throughput data is listed in Supplementary Figure 2a. And the genome coverage wiggles were generated with BEDTools [8]. The other RNA-Seq data of human tissues, cell types and cancer cell types were retrieved from Illumina Human Body Map Project [4] and human ENCODE project [9]. For identifying the novel lincRNA transcripts, the single-exon transcripts were eliminated from the Cufflinks-assembled transcriptome[4]. De novo transcript assembly was processed through Trinity [10]. Coding potential scores were assessed by PhyloCSF [11] and CPAT [12].

Document S2: Coding potential analysis

We employed the online CPAT server [12] (http://lilab.research.bcm.edu/cpat/) and PhyloCSF [11] to assess the coding potential of the long noncoding RNA. The CPAT result are as follows. For PhyloCSF analysis, we obtained the 46-way multi-alignment fasta sequences for Epist and using the ‘Stitch Gene blocks’ tools on the Galaxy. The coding potential was evaluated with any of the 3 reading frames. Scores are measured in decibans and represent the likelihood ratio which a sequence is protein-coding rather than noncoding. The score of Epist is 26.9283, lower than the

protein-coding cut-off (50) [13].

Coding Potential of long noncoding RNAs calculated by CPAT.

Sequence Name	RNA Size	ORF Size	Ficket Score	Hexamer Score	Coding Probability	Coding Label
Epist	582	315	0.9149	0.0626319698199	0.19179398202379	no

Document S3: Search for novel lincRNA transcripts and gene fusions in ESCC

In contrast to microarrays, RNA-seq can be employed to identify the novel transcripts across the genome. We used the single-end RNA-seq data (GSE29968) to identify novel lincRNA candidates according to the pipeline described in Materials and Methods (Figure S1B), and identified 31 novel lincRNA candidates (Table S7). The expression profile of these novel lincRNAs across the 3 ESCC paired tissues are given in Table S7.

Also, RNA-seq data can be used to find the gene fusions and has already led to the discovery of several gene fusions in some cancers [14], such as the *BCR-ABL1* fusion in K562 cell line. We employed the above RNA-seq data to detect expressed gene fusions. However, no recurrent gene fusions were identified in these data. The depth of the RNA-seq data we analyzed is comparable to that in a work identifying recurrent rearrangements of *CIITA* in Hodgkin lymphoma cell lines [15]. On the other hand, gene fusions were neither detected in an analysis on the Sézary syndrome [16]. Although we do not observe the gene fusions in ESCC, we still cannot rule out the possibilities that the fusion transcripts exist, such as the heterologous gene linking to an oncogene.

Fusion transcripts discovery

The FusionMap software (version: 6.0.0) [17] was used with default parameters to detect the fusion transcripts from Esophageal Squamous Cell Carcinoma (ESCC) as well as the normal tissue from each patient. PCR was performed on cDNA libraries from the ESCC tissues to confirm the presence of possible fusion transcripts.

References:

1. Ma S, Bao JYJ, Kwan PS, Chan YP, Tong CM, Fu L, Zhang N, Tong AHY, Qin YR, Tsao SW, Chan KW, Lok S and Guan XY. Identification of PTK6, via RNA Sequencing Analysis, as a Suppressor of Esophageal Squamous Cell Carcinoma. Gastroenterology. 2012; 143(3):675-686.e612.

2. Tong M, Chan KW, Bao JYJ, Wong KY, Chen J-N, Kwan PS, Tang KH, Fu L, Qin Y-R, Lok S, Guan X-Y and Ma S. Rab25 Is a Tumor Suppressor Gene with Antiangiogenic and Anti-Invasive Activities in Esophageal Squamous Cell Carcinoma. Cancer Res. 2012; 72(22):6024-6035.
3. Harrow J, Frankish A, Gonzalez JM, Tapanari E, Diekhans M, Kokocinski F, Aken BL, Barrell D, Zadissa A, Searle S, Barnes I, Bignell A, Boychenko V, Hunt T, Kay M, Mukherjee G, et al. GENCODE: The reference human genome annotation for The ENCODE Project. Genome Res. 2012; 22(9):1760-1774.
4. Cabili MN, Trapnell C, Goff L, Koziol M, Tazon-Vega B, Regev A and Rinn JL. Integrative annotation of human large intergenic noncoding RNAs reveals global properties and specific subclasses. Genes Dev. 2011; 25(18):1915-1927.
5. Trapnell C, Williams BA, Pertea G, Mortazavi A, Kwan G, van Baren MJ, Salzberg SL, Wold BJ and Pachter L. Transcript assembly and quantification by RNA-Seq reveals unannotated transcripts and isoform switching during cell differentiation. Nat Biotechnol. 2010; 28(5):511-515.
6. Tusher VG, Tibshirani R and Chu G. Significance analysis of microarrays applied to the ionizing radiation response. Proceedings of the National Academy of Sciences of the United States of America. 2001; 98(9):5116-5121.
7. Anders S, McCarthy DJ, Chen Y, Okoniewski M, Smyth GK, Huber W and Robinson MD. Count-based differential expression analysis of RNA sequencing data using R and Bioconductor. Nat Protoc. 2013; 8(9):1765-1786.
8. Quinlan AR and Hall IM. BEDTools: a flexible suite of utilities for comparing genomic features. Bioinformatics. 2010; 26(6):841-842.
9. Dunham I, Kundaje A, Aldred SF, Collins PJ, Davis CA, Doyle F, Epstein CB, Frietze S, Harrow J, Kaul R, Khatun J, Lajoie BR, Landt SG, Lee BK, Pauli F, Rosenbloom KR, et al. An integrated encyclopedia of DNA elements in the human genome. Nature. 2012; 489(7414):57-74.
10. Haas BJ, Papanicolaou A, Yassour M, Grabherr M, Blood PD, Bowden J, Couger MB, Eccles D, Li B, Lieber M, Macmanes MD, Ott M, Orvis J, Pochet N, Strozzi F, Weeks N, et al. De novo transcript sequence reconstruction from RNA-seq using the Trinity platform for reference generation and analysis. Nat Protoc. 2013; 8(8):1494-1512.
11. Lin MF, Jungreis I and Kellis M. PhyloCSF: a comparative genomics method to distinguish protein coding and non-coding regions. Bioinformatics. 2011; 27(13):i275-282.
12. Wang L, Park HJ, Dasari S, Wang S, Kocher JP and Li W. CPAT: Coding-Potential Assessment Tool using an alignment-free logistic regression model. Nucleic Acids Res. 2013; 41(6):e74.
13. Guttman M, Russell P, Ingolia NT, Weissman JS and Lander ES. Ribosome Profiling Provides Evidence that Large Noncoding RNAs Do Not Encode Proteins. Cell. 2013; 154(1):240-251.
14. Maher CA, Kumar-Sinha C, Cao X, Kalyana-Sundaram S, Han B, Jing X, Sam L, Barrette T, Palanisamy N and Chinnaiyan AM. Transcriptome sequencing to detect gene fusions in cancer. Nature. 2009; 458(7234):97-101.
15. Steidl C, Shah SP, Woolcock BW, Rui L, Kawahara M, Farinha P, Johnson NA, Zhao Y, Telenius A, Neriah SB, McPherson A, Meissner B, Okoye UC, Diepstra A, van den Berg A, Sun M, et al. MHC class II transactivator CIITA is a recurrent gene fusion partner in lymphoid cancers. Nature. 2011; 471(7338):377-381.
16. Lee CS, Ungewickell A, Bhaduri A, Qu K, Webster DE, Armstrong R, Weng WK, Aros CJ, Mah A, Chen RO, Lin M, Sundram U, Chang HY, Kretz M, Kim YH and Khavari PA. Transcriptome sequencing in Sezary syndrome identifies Sezary cell and mycosis fungoides-associated lncRNAs and novel transcripts. Blood. 2012; 120(16):3288-3297.
17. Ge H, Liu K, Juan T, Fang F, Newman M and Hoeck W. FusionMap: detecting fusion genes from next-generation sequencing data at base-pair resolution. Bioinformatics. 2011; 27(14):1922-1928.
