# Supplementary material for: Transcriptome profiling of esophageal squamous cell carcinoma reveals a long noncoding RNA acting as a tumor suppressor
Source: Oncotarget. 2015 May 19;6(19):17065–80. doi: 10.18632/oncotarget.4185 (PMC4627292; doi:10.18632/oncotarget.4185)
Supplement: Supplementary file 10 [file oncotarget-06-17065-s010.docx]

**Primers used in this work**

**Protein-coding genes primers (direction: 5’-> 3’)**

>PTK6_F AACACATCCTGGCGCTGTA

>PTK6_R CTGCGACTCCAGGTAACACA

>Rab25_F GCTGCTGTCAAGGCTCAGAT

>Rab25_R GCATAGGTCTGGTGCTTGGT

>PITX1_F GTCGTCTGACACGGAGCTG

>PITX1_R AACGTGGCCTCTAGCTCTTG

>RASAL1_F CAGAAGTGCAGGGTGAGATC

>RASAL1_R GTCAGATGTGCCAGAGATGTC

>TERT_F AGAGTGTCTGGAGCAAGTTG

>TERT_R GATGAAGCGGAGTCTGGAC

**Northern Blotting Probe Primers**

(red nucleotides indicated T7 promoter sequences)

>Epist_F3 GGAGAAGTGGGCAGGAGAGAG
>Epist_R3 TAATACGACTCACTATAGGGCATTGCTGCCCGAACGAACG

**Long noncoding RNAs primers (direction: 5’-> 3’)**

>XLOC_012538_F GCAGGGTAAGGACAGGTAAAA

>XLOC_012538_R GGGATGACGATCTTAGGGAAAG

>NR_024386_F ATGGCCTGATGGAGTGCTAC

>NR_024386_R ATCTTATGGCCCGAGTGATG

>NR_033961_F AAGGATATTGTGGCTGGAGAC

>NR_033961_R CTGTAGGAAGGCAATCTGGG

>XLOC_004273_F TGGAACTGCCTTCTTTACTGCTC

>XLOC_004273_R ATCGCAGGTCATCAAGTGGTATT

>ENST00000478294.1_F AGAAGACAGTGGGTGAAGTCCTG

>ENST00000478294.1_R GTGAAGGGCTGAAGGGTTTAGAT

>H19_F AGCGGGTCTGTTTCTTTACTTC

>H19_R AGGTAGTGCAGTGGTTGTAAAG

>Epist_F CGCGGGAATGTCTTTATTGG

>Epist_R GCACAGGTCGGGCTTTT

>HOTAIR_F AGAGAAAAGGCTGAAATGGAGG

>HORAIR_R AGGTCGGTACTGGCTTAGG

**Oligos used in this work (r for RNA and d for DNA)**

si-PITX1-1-sense

rUrCrArGrCrArCrCrCrArGrCrUrCrCrArUrCrUrCrCrUrCdCdA

si-PITX1-1-antisense

rUrGrGrArGrGrArGrArUrGrGrArGrCrUrGrGrGrUrGrCrUrGrArGrA

si-PITX1-2-sense

rArCrUrUrCrArCrArArGrCrCrArGrCrArGrUrUrGrCrArAdGdA

si-PITX1-2-antisense

rUrCrUrUrGrCrArArCrUrGrCrUrGrGrCrUrUrGrUrGrArArGrUrGrC

si-Epist-1-sense

rCrUrUrUrArUrUrGrGrArCrGrUrUrArCrGrArArGrGrGrAdAdT

si-Epist-1-antisense

rArUrUrCrCrCrUrUrCrGrUrArArCrGrUrCrCrArArUrArArArGrArC

siEpist-2-sense

rArArCrGrArArCrGrArArCrGrArArCrGrArArCrGrArArGdCdG

siEpist-2-antisense

rCrGrCrUrUrCrGrUrUrCrGrUrUrCrGrUrUrCrGrUrUrCrGrUrUrCrG
